# Integrated analyses highlight interactions between the three-dimensional genome and DNA, RNA and epigenomic alterations in metastatic prostate cancer

**DOI:** 10.1038/s41588-024-01826-3

**Published:** 2024-07-17

**Authors:** Shuang G. Zhao, Matthew Bootsma, Stanley Zhou, Raunak Shrestha, Thaidy Moreno-Rodriguez, Arian Lundberg, Chu Pan, Christopher Arlidge, James R. Hawley, Adam Foye, Alana S. Weinstein, Martin Sjöström, Meng Zhang, Haolong Li, Lisa N. Chesner, Nicholas R. Rydzewski, Kyle T. Helzer, Yue Shi, West Coast Dream Team Consortium, Adina M. Bailey, Li Zhang, Tomasz M. Beer, George Thomas, Kim N. Chi, Martin Gleave, Amina Zoubeidi, Robert E. Reiter, Matthew B. Rettig, Owen Witte, Rohit Bose, Franklin W. Huang, Larry Fong, Primo N. Lara, Christopher P. Evans, Jiaoti Huang, Molly Lynch, Scott M. Dehm, Joshua M. Lang, Joshi J. Alumkal, Hansen H. He, Alexander W. Wyatt, Rahul Aggarwal, Wilbert Zwart, Eric J. Small, David A. Quigley, Mathieu Lupien, Felix Y. Feng

**Affiliations:** 1 Department of Human Oncology, University of Wisconsin-Madison, Madison, WI USA; 2 Carbone Cancer Center, University of Wisconsin-Madison, Madison, WI USA; 3 William S. Middleton Memorial Veterans Hospital, Madison, Madison, WI USA; 4 Princess Margaret Cancer Centre, University Health Network, Toronto, Ontario Canada; 5 Department of Medical Biophysics, University of Toronto, Toronto, Ontario Canada; 6 Department of Radiation Oncology, University of California San Francisco, San Francisco, CA USA; 7 Helen Diller Family Comprehensive Cancer Center, University of California San Francisco, San Francisco, CA USA; 8 Division of Hematology and Oncology, Department of Medicine, University of California San Francisco, San Francisco, CA USA; 9 Radiation Oncology Branch, National Cancer Institute, National Institutes of Health, Bethesda, MD USA; 10 Masonic Cancer Center, University of Minnesota, Minneapolis, MN USA; 11 Department of Laboratory Medicine and Pathology, University of Minnesota, Minneapolis, MN USA; 12 Department of Urology, University of Minnesota, Minneapolis, MN USA; 13 Department of Medicine, University of Wisconsin-Madison, Madison, WI USA; 14 Department of Internal Medicine, Division of Hematology-Oncology, University of Michigan Rogel Cancer Center, Ann Arbor, MI USA; 15 Department of Urologic Sciences, Vancouver Prostate Centre, University of British Columbia, Vancouver, British Columbia Canada; 16 Michael Smith Genome Sciences Centre, BC Cancer, Vancouver, British Columbia Canada; 17 Netherlands Cancer Institute, Oncode Institute, Amsterdam, the Netherlands; 18 Department of Epidemiology and Biostatistics, University of California San Francisco, San Francisco, CA USA; 19 Department of Urology, University of California San Francisco, San Francisco, CA USA; 20 OHSU Knight Cancer Institute, Portland, OR USA; 21 BC Cancer, Vancouver, British Columbia Canada; 22 University of California Los Angeles, Los Angeles, CA USA; 23 VA Greater Los Angeles Healthcare System, Los Angeles, CA USA; 24 Department of Microbiology, Immunology and Molecular Genetics at the David Geffen School of Medicine, University of California Los Angeles, Los Angeles, CA USA; 25 Division of Hematology and Oncology, Department of Internal Medicine, University of California Davis, Sacramento, CA USA; 26 Comprehensive Cancer Center, University of California Davis, Sacramento, CA USA; 27 Department of Urologic Surgery, University of California Davis, Sacramento, CA USA; 28 Department of Pathology, Duke University, Durham, NC USA

**Keywords:** Prostate cancer, Prostate cancer

## Abstract

The impact of variations in the three-dimensional structure of the genome has been recognized, but solid cancer tissue studies are limited. Here, we performed integrated deep Hi-C sequencing with matched whole-genome sequencing, whole-genome bisulfite sequencing, 5-hydroxymethylcytosine (5hmC) sequencing and RNA sequencing across a cohort of 80 biopsy samples from patients with metastatic castration-resistant prostate cancer. Dramatic differences were present in gene expression, 5-methylcytosine/5hmC methylation and in structural variation versus mutation rate between A and B (open and closed) chromatin compartments. A subset of tumors exhibited depleted regional chromatin contacts at the *AR* locus, linked to extrachromosomal circular DNA (ecDNA) and worse response to *AR* signaling inhibitors. We also identified topological subtypes associated with stark differences in methylation structure, gene expression and prognosis. Our data suggested that DNA interactions may predispose to structural variant formation, exemplified by the recurrent *TMPRSS2*–*ERG* fusion. This comprehensive integrated sequencing effort represents a unique clinical tumor resource.

## Abstract (web summary)

A multiomic approach profiles the three-dimensional, epigenetic and mutational landscapes of 80 metastatic prostate cancer biopsies. Hi-C experiments identify an extrachromosomal circular DNA at the *AR* locus associated with therapy resistance.

## Main

The advances in next-generation sequencing (NGS) of the past two decades have expanded our understanding of the genomic and epigenomic alterations that drive oncogenesis. The study of prostate cancer exemplifies this trend across cancers. Through large-scale efforts such as The Cancer Genome Atlas^1^, the landscape of genomic, transcriptomic and epigenomic alterations across primary prostate cancer has been extensively cataloged^2,3^. Because of the logistical and technical challenges of profiling metastatic biopsy specimens, only recently researchers have been able to integrate techniques such as whole-exome sequencing and whole-genome sequencing (WGS), RNA sequencing (RNA-seq), whole-genome bisulfite sequencing (WGBS), 5-hydroxymethylcytosine (5hmC) sequencing and chromatin immunoprecipitation followed by NGS (ChIP–seq) in metastatic castration-resistant prostate cancer^4–12^ (mCRPC), the final and lethal stage of the most common malignancy in men. DNA sequencing provides only one-dimensional information on the order of bases. Epigenomic changes, such as DNA 5-methylcytosine (5mC) and 5hmC methylation, and histone modifications add an important second dimension in understanding the dysregulation of genes present in cancer.

Despite these remarkable advances in our understanding of the linear cancer genome, we lack a clear understanding how the three-dimensional (3D) conformation of the genome, a critical regulator of cellular programs^13,14^, influences human malignancy. With the advancement of genome-wide chromatin conformation capture technologies, such as Hi-C^15^, we can begin to understand how distinct types of tertiary structures in the cancer genome influence tumor cells. A and B compartments are regions of chromatin that are open (A) or closed (B), influencing gene expression^15^. Topologically associated domains (TADs) are regions of the genome enriched for intradomain interactions^16^. Finally, individual DNA loops can bring distant parts of the genome together, such as enhancers and genes, to drive expression^17^. In addition to the 3D structure of the genome, Hi-C has also been shown to identify structural variants (SVs) in cancer, complementing analysis by WGS^18^. Integration of Hi-C with standard NGS approaches can provide insights into how compartment biology and DNA topology influence and interact with somatic alterations in cancer.

Despite the potential of leveraging Hi-C to expand our understanding of the 3D genome globally, this method normally requires large amounts of input tissue and thus has primarily been performed in the context of cell lines as part of the ENCODE project^19^. Low-input versions of Hi-C have since been developed to facilitate its use in cancer tissue studies^18,20^. However, to date, only a handful of Hi-C studies have been performed using human tumor tissue. Studies have profiled a small number of tumor samples from diffuse large B cell lymphoma^20^ (*n* = 1), lung cancer^21^ (*n* = 2), nasopharyngeal cancer^22,23^ (*n* = 3 and *n* = 6), diffuse intrinsic pontine glioma^24^ (*n* = 1), T cell acute lymphoblastic leukemia^25,26^ (*n* = 8 and *n* = 18), primary prostate cancer^18^ (*n* = 12), primary colon cancers^27^ (*n* = 26) and more recently acute myelogenous leukemia^28^ (*n* = 25). Because of these small numbers, especially for solid tumors, most of our insights into the 3D genome of cancer come from in vitro model systems rather than clinical patient samples.

In this study, we performed low-input Hi-C on 80 patients with metastatic prostate cancer and provided integrated analysis with deep WGS, WGBS, 5hmC-seq and RNA-seq in any cancer. We identified relationships between A and B compartments, TADs, mutations, SVs, 5mC and 5hmC methylation, and gene expression that more fully describe the complex regulatory structures of cancer.

## Results

### Integration of Hi-C, WGS, WGBS, RNA-seq and 5hmC-seq across 80 mCRPC metastatic biopsies

**Fig. 1:**
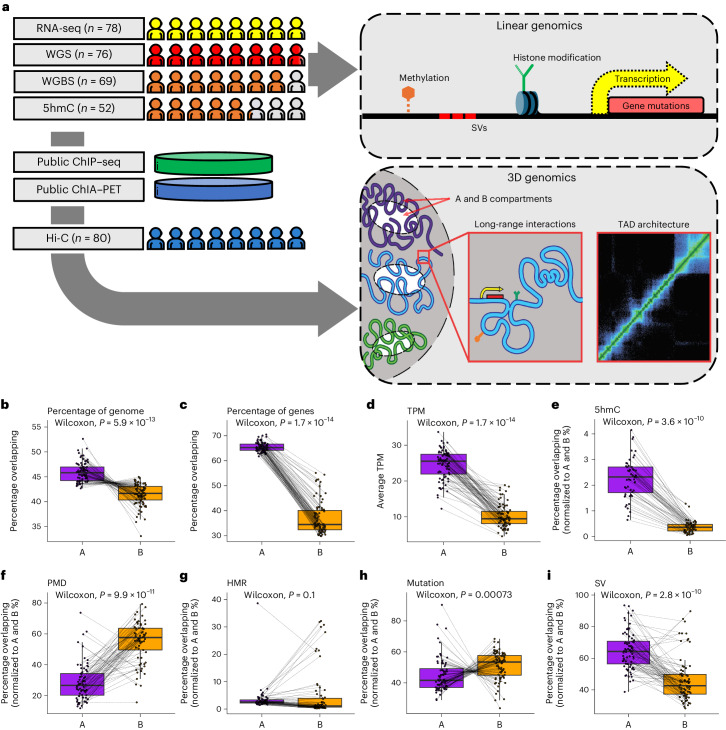
Overview of the 3D multiomic approach. **a**, Study schematic. **b**–**i**, A and B compartment associations with the percentage of the genome covered (*n* = 80) (**b**), the percentage overlapping with genes (*n* = 80) (**c**), the mean transcripts per million (TPM) of the genes (*n* = 78) (**d**), the percentage overlapping with the 5hmC peaks (*n* = 52) (**e**), the percentage overlapping with PMDs (*n* = 69) (**f**), the percentage overlapping with HMRs (*n* = 69) (**g**), the percentage overlapping with mutations (*n* = 76) (**h**) and the percentage overlapping with SVs (*n* = 76) (**i**). Each point represents one sample. The dotted lines connect the values from the A and B compartments in the same sample. *P* values were computed using a paired, two-sided Wilcoxon rank-sum test. The center line represents the median; the box limits represent the upper and lower quartiles; and the whiskers represent 1.5× the interquartile range (IQR). Source data

To define the 3D organization of the genome in mCRPC, 80 metastatic biopsy samples from patients with mCRPC from the Stand Up 2 Cancer (SU2C) - Prostate Cancer Foundation (PCF) West Coast Dream Team (WCDT) consortium were processed using a low-input Hi-C assay^18^. Most patients in this cohort received one or more previous treatments for mCRPC before their biopsy. Sixty-two of 80 (77.5%) were previously treated with an androgen signaling inhibitor (ASI) (for example, abiraterone, enzalutamide); 12 of 80 (15%) were previously treated with chemotherapy. Finally, 12 of 80 (15%) were previously treated with sipileucel-T (Supplementary Table 1). Deep Hi-C sequencing to a median of approximately 1.3 billion reads per sample resulted in a 10-kb spatial resolution of chromatin interactions, which we integrated with deep WGS (*n* = 76, median depth 129×), RNA-seq (*n* = 78, median = 113 million reads per sample), deep WGBS (*n* = 69, median depth 46×) and 5hmC-seq (*n* = 52, median = 26 million reads per sample), all in the same cohort of samples (Fig. 1a and Supplementary Table 1).

### A and B compartments are associated with somatic DNA alterations, gene expression and methylation

Active chromatin loops, engaging regulatory elements in active gene transcription, form A compartments, while repressive loops cluster into B compartments^15^. In mCRPC, we found that the A compartments cover more of the genome than the B compartments (Fig. 1b; *P* < 0.001), which we normalized for subsequently. Open chromatin in A compartments was associated with disproportionately higher gene density and higher gene expression than closed chromatin in B compartments (Fig. 1c,d; *P* < 0.001), as expected. We also observed an increase in 5hmC, a marker of active transcription^6^, in A compartments (Fig. 1e; *P* < 0.001). DNA 5mC methylation is a critical regulator of the 3D genome^29,30^ and we observed marked differences in the percentage of A and B compartments overlapping partially methylated domains (PMDs) (Fig. 1f; *P* < 0.001), which is consistent with previous in vitro data^31^, but not hypomethylated regions (HMRs) (Fig. 1g; *P* = 0.1). At the nuclear periphery, chromatin is organized in lamina-associated domains (LADs), which are involved in the spatiotemporal regulation of replication and transcription^32,33^. Both PMDs^32^ and B compartments^33^ were associated with LADs, providing a probable explanation for the observed association. A compartments harbor fewer mutations in tumors compared to B compartments, an observation that may be explained by the increased accessibility of open chromatin to DNA repair proteins^31,34–36^. Consistent with these previous studies, we observed that the global mutational burden was significantly lower in A compartments compared to B compartments (Fig. 1h; *P* < 0.001). In contrast, SV frequency was significantly higher in A compartments compared to B compartments (Fig. 1i; *P* < 0.001). This finding was consistent with the suggested model for decreased mutations in A compartments, in that the formation of an SV requires double-stranded DNA break repair; increased accessibility in A compartments would result in higher apparent SV rates. Collectively, our global analysis on A and B compartment composition in mCRPC supports associations between open A compartments and elevated gene expression and reveals a relationship between compartments and the type of genetic variants, where SVs and mutations are enriched in A and B compartments, respectively.

### Hi-C identifies AR extrachromosomal DNA

**Fig. 2:**
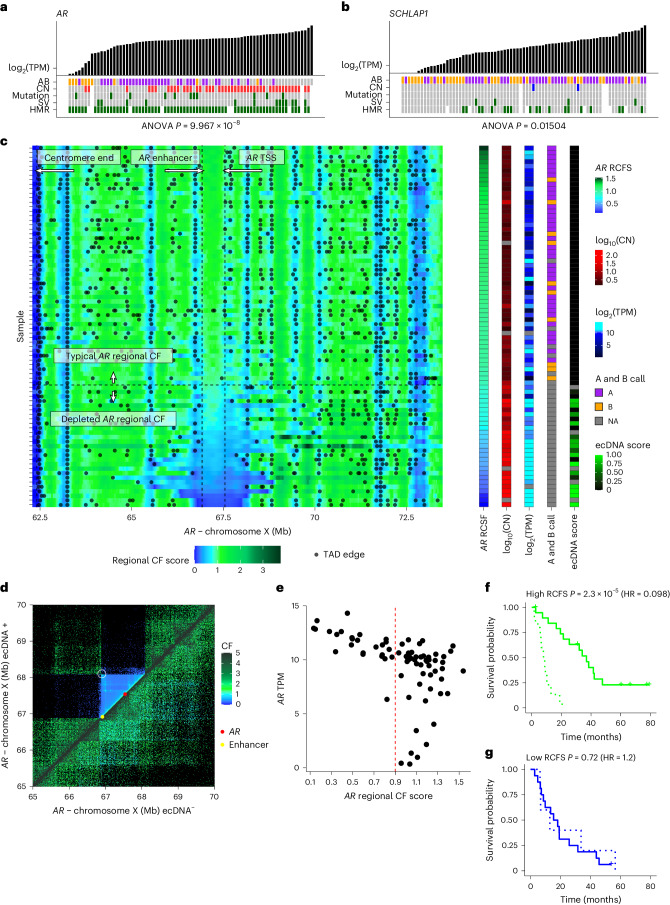
AR ecDNA. **a**,**b**, Gene expression versus genomic alterations (CN gain, red; loss, blue). Promoter methylation for *AR* (**a**) and *SChLAP1* (**b**). **c**, RCFS, that is, average contact frequency within a sliding window applied to the Hi-C contact matrix for each sample (row) showing a depletion of local contacts (low RCFS) surrounding the *AR* locus in a subset of samples, which were enriched for samples with ecDNA events detected by WGS at this region. The local TAD structure (black points indicating the TAD edges) is annotated. Right, the corresponding sample meta-data includes the average RCFS between *AR* enhancer and transcription start site (TSS), CN, *AR* TPM, A and B domain call, and the degree of cyclical amplification (indicating ecDNA) from AmpliconArchitect. Tiles with a gray fill represent values not available. **d**, Bin pair contact frequencies surrounding the *AR* locus for a single ecDNA^+^ sample above the diagonal and a single ecDNA^−^ sample below the diagonal, further illustrating the contrast in contact frequency. The region circled in white shows an increased interaction between the two ends of the ecDNA region, which is consistent with circularization. **e**, Per-sample *AR* TPM versus RCFS; the red dashed line is the same cutoff for categorizing samples as depleted versus typical *AR* RCFS as in **c**. **f**,**g**, Kaplan–Meier curves showing OS as a function of RCFS (high in **f**, low in **g**) and whether an ASI was used as the next-line therapy after biopsy (solid line, true; dotted line, false; interaction *P* = 0.0051). HR, Cox hazard ratio. ANOVA, analysis of variance. Source data

Extrachromosomal circular DNA (ecDNA) from frequently amplified oncogenes and associated regulatory elements is common across cancers, accentuating oncogenic properties^37,38^. The androgen receptor (*AR*) gene lies in the most frequently amplified locus in mCRPC^4,11,12^. The A and B compartment assignment of the *AR* locus was significantly associated with *AR* gene expression even after accounting for other genomic and epigenomic alterations (*P* < 0.001 for *AR*; Fig. 2a). However, there were cases where A and B compartment status could not be ascertained for the *AR* locus. We observed that this coincided with samples harboring very high *AR* expression and copy number (CN) amplification. As a control, we compared A and B compartment assignments at *SChLAP1*, a prostate cancer-specific long noncoding RNA associated with prostate cancer progression both in vitro^39^ and clinically^40^, which is not frequently amplified in mCRPC. Assignment of the *SChLAP1* loci to the A and B compartments occurred in nearly all samples and was significantly associated with expression (*P* = 0.015 for *SChLAP1*; Fig. 2b). Focusing on the samples lacking an A or B compartment assignment, we next examined the regional contact frequency sliding-window (RCFS) score at the *AR* locus. This score summarizes 3D Hi-C information as a single dimension and is primarily used to identify distinct TADs. In most samples, typical RCFS scores at the *AR* locus were consistent with frequent contact between *AR* and adjacent genomic loci on chromosome X. However, we observed a dramatic decrease in RCFS values around the *AR* locus in samples corresponding with absent A and B calls, frequently also including the upstream enhancer locus (Fig. 2c). In these 27 samples, low RCFS values suggested an unexpected lack of contact between the *AR* locus and nearby DNA (Fig. 2d). This reduced contact (and therefore proximity) combined with *AR* amplification (which typically causes increased, not decreased contact frequencies^41^) and elevated expression (Fig. 2e), were suggestive of ecDNA-mediated oncogene activation^42,43^. Furthermore, we observed an increased Hi-C interaction between the two ends of an ecDNA region, which is consistent with circularization^42^ (Fig. 2d). To confirm this hypothesis, we examined WGS from the same samples using AmpliconArchitect, a tool designed to identify ecDNA and other complex structural rearrangements^37^. Most samples with a low RCFS score (77%) had evidence for ecDNA using AmpliconArchitect (17 of 22 samples where data were available). The AmpliconArchitect results further supported evidence for diverse combinations of both simple linear and nonlinear amplicons and complex rearrangements expected from breakage–fusion–bridge (BFB) cycles (Supplementary Table 2) or even involving segments of multiple chromosomes (Supplementary Fig. 1). None of the typical RCFS samples had any evidence for ecDNA using AmpliconArchitect. We performed FISH on three mCRPC samples identified as *AR* ecDNA-positive by AmpliconArchitect and observed widespread *AR* staining, which was absent in three samples without support for *AR* ecDNA from AmpliconArchitect (Supplementary Fig. 2). While deep WGS is better suited for characterizing the exact rearrangement that is occurring, Hi-C may provide complementary information on the 3D conformation of these rearrangements, especially the presence of ecDNA, as quantified by the RCFS.

By carrying and amplifying oncogenes, ecDNAs are often associated with poor outcomes across cancer types^37^. As ASIs are the backbone systemic treatment in metastatic prostate cancer, and *AR* gene amplification is a potential resistance mechanism, we hypothesized that depleted *AR* locus RCFS (enriched for ecDNA) would portend a decreased benefit from treatment with ASIs. Examining the clinical data from our cohort^44^, we found that patients with a typical *AR* RCFS had a significant benefit to overall survival (OS) when their treatment after biopsy was an ASI (*P* < 0.0001, hazard ratio (HR) = 0.098; Fig. 2f), while there was no benefit to ASI in patients with a depleted RCFS (*P* = 0.72; Fig. 2g). The Cox regression interaction between ASI treatment and RCFS (measured as a continuous score) was statistically significant (*P* = 0.0051), suggesting that the *AR* locus RCFS predicts a diminished benefit of ASI treatment. The interaction was not significant when we repeated the same analysis with the *AR* CN, indicating that this phenomenon was not solely due to *AR* amplification. Collectively, our results revealed that mCRPC tumors with ecDNA amplifying *AR* may be more refractory to ASI therapy.

### 3D interactions between genes and enhancers are associated with gene expression

**Fig. 3:**
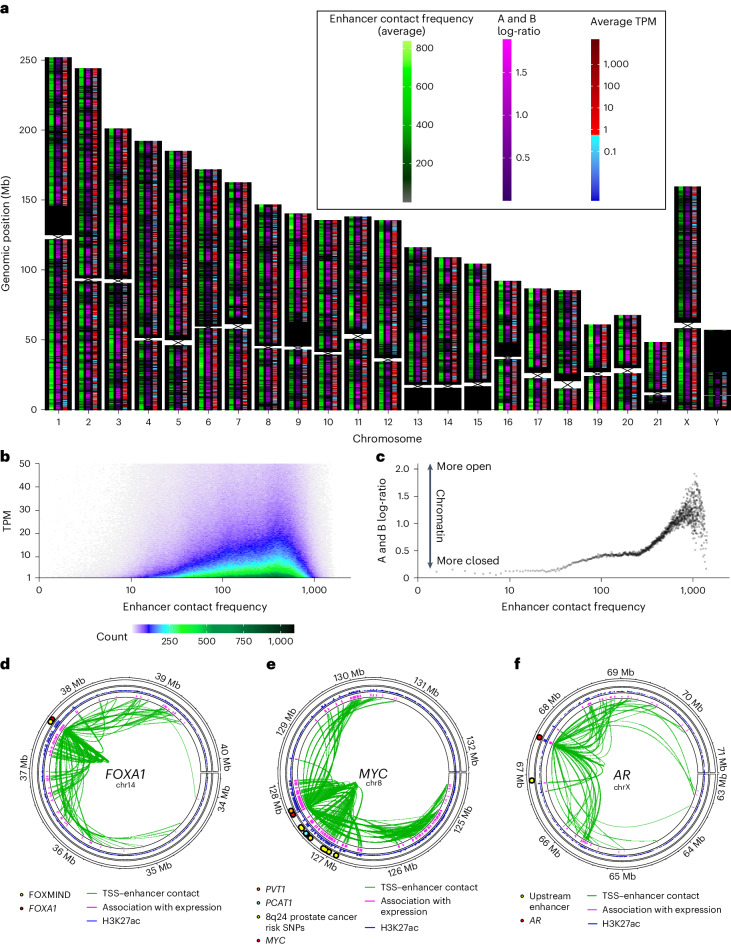
Genome-wide analysis of gene–enhancer contact. **a**, Chromosomes with three tracks plotted for 10-kb bins. Left to right, within each chromosome, the tracks represent the sum of 3D contact frequencies of a bin with all enhancer regions on the same chromosome (averaged across samples); the log-ratio of A versus B compartment calls across samples at that bin, where higher values imply more open chromatin (A compartment) and lower values imply more closed chromatin (B compartment); and the average TPM of genes whose TSS falls in the given bin. **b**, Two-dimensional density plot showing how the contact frequency of 3D chromatin interactions between a gene’s TSS and enhancers (column 1, **a**) relates to expression (column 3, **a**). **c**, Scatter plot showing the relationship between the A and B compartment log-ratio (column 2, **a**) and the contact frequency of 3D contacts with enhancers (column 1, **a**). **d**–**f**, Recurrent intrachromosomal contacts (green links, the height represents the frequency of interaction observed across samples) between the TSS (red dot) of *FOXA1* (**d**), *MYC* (**e**) and *AR* (**f**), and putative enhancers (blue peaks around the outside, recurrently hypomethylated H3K27ac regions, with peak height equal to the number of hypomethylated samples). The association between putative enhancers and gene expression was measured using a *t*-statistic between the hypomethylation status of enhancer and gene expression (pink peaks around the outside); all *P* < 0.05. All *t*-statistics were positive. Source data

To investigate how Hi-C could elucidate the effects of regulatory regions linked through 3D DNA structure to the promoters of genes, we integrated our Hi-C data with linear prostate cancer histone modification ChIP–seq data from previously published datasets^10^. We expected that higher levels of chromatin interaction between gene promoters and enhancers would be associated with higher gene expression levels. To test this, we identified chromatin loops connecting gene promoters to regulatory regions harboring candidate enhancers (marked by H3K27ac and recurrent hypomethylation) and examined the strength of these 3D chromatin interactions (Fig. 3a). Genome-wide gene expression increased as the interaction strength with regions harboring enhancers increased, but only up to a point, after which gene expression decreased (Fig. 3b). An increase in closed, and therefore more tightly interacting, but transcriptionally inactive chromatin may account for this inflection. We observed evidence for this in the A:B ratio that increasingly favors open A compartments up to around the same point, but then diverges (Fig. 3c). The importance of chromatin interactions with regulatory regions is well characterized, and our data support the role of the 3D genome as expected.

We next examined three key prostate cancer oncogenes known to have regional enhancer interactions: *FOXA1*, *MYC* and *AR*^4,5,45–49^. Recurrent contacts between the gene and enhancer regions (as above) were localized to a several megabase-sized region around each gene (Fig. 3d–f). The FOXMIND^46^ enhancer for *FOXA1* showed evidence of recurrent chromatin looping, with hypomethylation correlated with *FOXA1* gene expression. However, there were also multiple other putative enhancer regions (based on H3K27ac and recurrent hypomethylation), which displayed interactions with *FOXA1*. Hypomethylation at these putative enhancers was correlated with *FOXA1* expression (Fig. 3d), with the degree of correlation diminishing as the enhancer distance from *FOXA1* increased (Supplementary Fig. 3). *MYC* has enhancers regionally^47^, within *PVT1* (refs. ^5,48^) and *PCAT1* (ref. ^49^). We saw evidence of recurrent chromatin looping from these enhancer regions to *MYC* (Fig. 3e). In addition, multiple prostate cancer risk loci have been described near *MYC* on chromosome 8q24 (ref. ^50^); we saw evidence of recurrent chromatin looping to many of these regions. Like *FOXA1*, there were also multiple other putative enhancer regions, with the degree of correlation with gene expression diminishing as the enhancer distance from *MYC* increases (Supplementary Fig. 3). The pattern was repeated around *AR*, with recurrent chromatin looping with the known upstream enhancer^4^, but also multiple other more distant putative enhancers, with the degree of correlation with gene expression diminishing as the enhancer distance from *AR* increases (Fig. 3f and Supplementary Fig. 3). Our data suggest a more complex *cis*-regulatory landscape than previously described in the literature for these key mCRPC oncogenes.

**Fig. 4:**
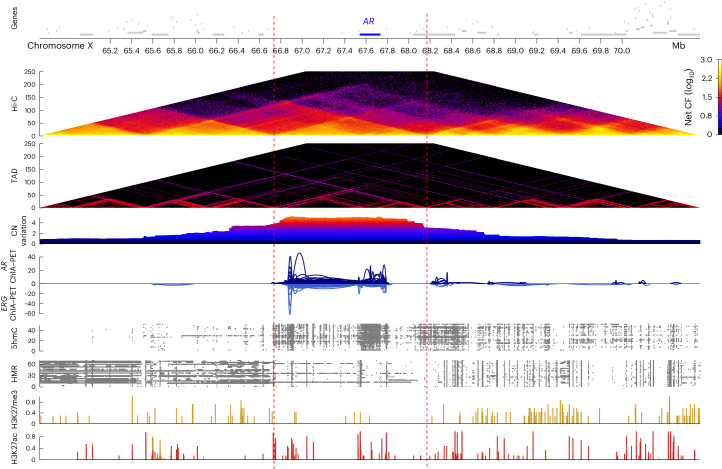
Genomic and epigenomic events surrounding *AR*. Top to bottom, genes (with *AR* in blue), net Hi-C contact frequency (log_10_) across our samples, TAD structure across our samples where the TADs of all samples are overlayed, average CN variation across our samples from WGS, published prostate cancer *ERG* and *AR* chromatin interaction analysis using paired-end tag sequencing (ChIA–PET), 5hmC peaks across our samples from 5hmC-seq, HMRs across our samples from WGBS and published prostate cancer ChIP–seq for H3K27ac. The red dashed lines indicate the border of TAD regions corresponding to common *AR* locus amplification borders. Source data

Chromatin loops cluster to form TADs such as those observed at the *AR* locus (Fig. 4). A plateau of amplification and 5hmC/hypomethylation of both the *AR* gene itself and its regulatory plexus of coamplified enhancers, is thought to be a critical driver of overexpression and resistance to therapies targeting *AR*^4,5,7,11,12^. This regulatory plexus is in the same TAD as the *AR* promoter in most samples. Interestingly, the amplified multi-enhancer region both upstream and downstream of *AR*^5^ frequently corresponded with the edges of TADs encompassing *AR*. The combination of the selective pressure of the dense cluster of *AR* enhancers combined with physical proximity because of the TAD structure of the *AR* locus may explain why the boundaries of the amplified region are so commonly conserved across mCRPC tumors, and illustrates how genome topology can potentially influence SVs, which we explore further below.

### 3D topology and structural variation patterns

**Fig. 5:**
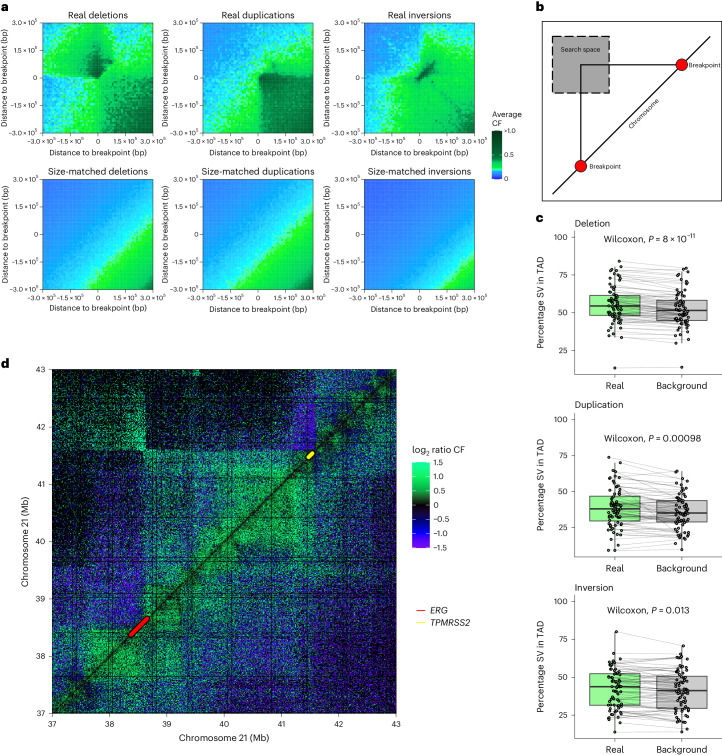
SVs and 3D organization of chromatin. **a**, Hi-C contact frequency signatures for three SV types (deletions, duplications, inversions). Signatures were defined by subsetting Hi-C contact frequencies in a 600-kb search space around each known SV detected using WGS, centered around the two breakpoints of the SV. The individual Hi-C submatrices for each SV in each sample were then averaged across each of the SV types (top) and compared to the size-matched background, where random regions of the same size as the SVs were visualized using the same approach as for the SVs (bottom). **b**, Schematic showing how the Hi-C contact frequency matrix was defined with respect to SV breakpoints, which were then averaged for all SVs across samples for **a**. **c**, Percentage of SVs versus the size-matched background where both ends were in the same TAD in the samples (*n* = 76 biologically independent samples). As we demonstrated the effect that SVs can have on the Hi-C interaction data, we instead examined TADs in samples other than the one with the SV to assess if the TAD structure potentially predisposes the formation of SVs. The center line represents the median; the box limits represent the upper and lower quartiles; the whiskers represent 1.5× the IQR. *P* values were computed using a two-sided, paired Wilcoxon rank-sum test. **d**, Median-centered log-ratio of Hi-C contact frequency comparing mCRPC samples and benign prostate. *TMPRSS2*–*ERG* fusion-positive samples (with breakpoints within five bins of the median) above the diagonal (canonical *TMPRSS2*–*ERG* fusion was defined using WGS, all but one consistent with monoallelic deletion, with one complex rearrangement) and negative below it. Green, higher in mCRPC; blue, higher in benign prostate. Source data

SVs are common across cancers, but especially in prostate cancer^4^. Different types of SVs result in varied genomic rearrangements that can alter gene expression programs. SVs are commonly identified using WGS, but also leave distinct patterns in Hi-C data because they alter which DNA regions are adjacent^18,51^. We identified clearly visible interactions in the Hi-C data where the two ends of the SVs identified using WGS are brought together (Fig. 5a,b). We did not see the same effect for the size-matched background, which consists of randomly generated locations across the genome of the same size distribution as real SVs. While physical proximity between two double-stranded DNA breaks is necessary, it is not sufficient for SV formation. Previous in vitro studies suggested that the proximity of chromosomal loci is important in radiation-induced rearrangements in human cells^52^ and mice^53^. We hypothesized that we might be able to see the influence of proximity on SV formation. Therefore, we took advantage of the fact that our dataset had paired WGS and Hi-C and examined the frequency with which actual SVs detected using WGS are within the bounds of the same TAD, compared to a size-matched background. We focused on deletions, duplications and inversions because these SVs have two breakpoints within the same chromosome. We found that for all three types of SVs, there was a significantly higher proportion of real SVs where both ends were in the same TAD compared to the size-matched background (Fig. 5c; *P* < 0.001 for deletions, *P* = 0.013 for inversions, *P* < 0.001 for duplications). While the effect was modest, the proximity of loci is only one factor among many in the formation of SVs and does not take into account the biological selective pressures favoring SVs in certain genomic locations. Altogether, our results suggest that TAD structure in prostate cancer is associated with the formation of SVs and supports the contribution of the 3D organization of the genome to the fundamental DNA alterations driving oncogenesis.

The *TMPRSS2*–*ERG* gene fusion is the most common recurrent fusion in prostate cancer, present in approximately half of tumors^4^. Previous experimental work demonstrated that androgen-induced colocalization of the *TMPRSS2* and *ERG* chromosomal loci in Lymph Node Carcinoma of the Prostate (LNCaP) cells increased the odds of forming this fusion in the presence of radiation^54^. Hi-C studies across both *TMPRSS2*–*ERG* fusion-negative and fusion-positive prostate cancer cell lines reported increased contact frequency in the region between *TMPRSS2* and *ERG* compared to benign prostate cell lines^55^. To validate these observations in samples from patients with mCRPC, we compared against five benign prostate samples and also found an increased contact frequency in this locus across *TMPRSS2*–*ERG-*positive and *TMPRSS2*–*ERG-*negative mCRPC tumors (Fig. 5d). The result was similar when comparing ten localized prostate cancers (five *TMPRSS2*–*ERG*^+^ and five *TMPRSS2*–*ERG*^−^) and benign prostate (Supplementary Fig. 4).

### TAD subtypes in mCRPC

**Fig. 6:**
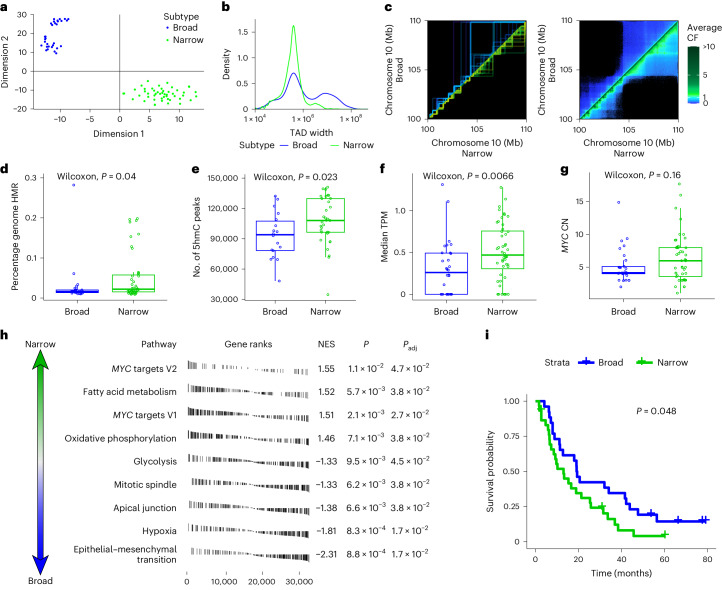
TAD subtypes. **a**, *t*-distributed stochastic neighbor embedding result defining two major subtypes of TAD architecture, defined from the TAD edges-per-megabase according to each sample. **b**, Distribution of the average per-sample TAD size according to subtype. **c**, Example of TAD structure across all samples, with broad-subtype samples above the axis and narrow-subtype samples below it. Likewise, average contact frequency between locations for the same region, illustrating the reduced level of 3D interaction in broad-subtype samples. **d**–**g**, Distributions between TAD subtypes for the proportion of the genome defined by HMRs (*n* = 69) (**d**), 5hmC peak abundance (normalized to read depth, *n* = 52) (**e**), median TPM (*n* = 78) (**f**) and distributions between TAD subtypes for *MYC* CN (*n* = 76) (**g**). The center line represents the median; the box represents the upper and lower quartiles; and the whiskers represent 1.5× the IQR. *P* values were computed using a two-sided Wilcoxon rank-sum test. **h**, Significantly enriched hallmark pathways as determined by gene set enrichment analysis, showing an enrichment for *MYC* targets in the narrow-subtype samples. (**i**) Kaplan–Meier curves showing OS. NES, normalized enrichment score. Source data

While elements of TAD structure were common across samples, there was also intersample variability as exemplified by the *AR* locus above (Fig. 2c). Therefore, we quantified the intrinsic patterns of TAD distributions across our cohort using the TAD edge density (per megabase) across the genome. We then visualized the distributions in two dimensions using *t*-distributed stochastic neighbor embedding (Fig. 6a). This analysis revealed two distinct tumor subgroups. One subgroup was characterized by broad TADs, the other by a narrow TAD structure within the broad TADs (Fig. 6b,c). We did not identify a significant association between broad versus narrow TAD structure tumors and tumor purity, ploidy, metastatic biopsy site or sequencing depth (Supplementary Fig. 5). This result was also not driven by sample processing batch or previous treatment exposure (Supplementary Fig. 6). There was also no association with whole-genome duplication, total SV abundance or the average or variance in CN per TAD (Supplementary Fig. 7). While we cannot exclude the contribution of other unmeasured covariates, when we integrated other sequencing modalities, we observed different methylation patterns between the broad and narrow TAD subgroups. The narrow subgroups had more HMRs (Fig. 6d; *P* = 0.04) and higher levels of 5hmC (Fig. 6e; *P* = 0.023). This corresponded to a difference in median gene expression levels between subgroups, with the narrow subgroup having higher median expression levels (Fig. 6f; *P* = 0.007) consistent with the increased HMR proportion (typically marking enhancers and promoters^5^) and increased 5hmC (marking actively transcribed genes^6^). Because the total normalized expression values (TPM) for all genes sums to a constant value within a sample, this difference probably reflects a difference in the global distribution of gene expression between TAD subtypes. We next performed gene set enrichment analysis to better understand differential gene expression between the broad and narrow subtype. We observed that the expression of *MYC* targets was strongly associated with the narrow subgroup (Fig. 6h); these samples were more likely to harbor amplified *MYC* (Fig. 6g; *P* = 0.16). These findings all suggest that the narrow subgroup had a different transcriptional phenotype, which was associated with worse OS (Fig. 6i; *P* = 0.048).

## Discussion

In this study, we profiled 80 metastatic castration-resistant prostate cancer biopsies, integrated with paired deep WGS, WGBS, RNA-seq and 5hmC-seq. The depth of our molecular evaluation was uniquely suited to assess the interaction between commonly observed genomic, epigenomic and transcriptomic alterations in cancer and the 3D topological structure of the genome. We describe several findings related to A and B compartments, global enhancer–gene interactions, ecDNA identification at the *AR* locus, TAD subtypes associated with DNA alterations, transcription, epigenetic and clinical differences, as well as relationships between the 3D prostate cancer genome and SV formation, especially for the highly recurrent *TMPRSS2*–*ERG* fusion and *AR* amplification.

With our broad sequencing, we demonstrate comprehensive associations with gene expression and methylation that are consistent with general A and B compartment biology^15,31–33^. In terms of cancer-specific phenomena, we identified associations with somatic mutations and SVs that logically follow from our understanding of A and B compartments and DNA repair^34–36^. Mapping the 3D organization of the cancer genome is imperative to understand aberrant gene expression programs that drive tumors. We also showed both globally, and for key oncogenes (*FOXA1*, *MYC*, *AR*), how DNA looping contributes to enhancer regulation. In addition to observing connectivity between these key oncogenes with known enhancers that have been functionally dissected previously^4,5,45–49^, we also identified a more complex regulatory structure with many more candidate enhancers that may contribute to regulating expression. The Hi-C interactome map combined with other modalities provides a more comprehensive view on how chromatin interactions can influence gene regulation and is probably not limited to specific oncogenes or mCRPC.

Structural variation is frequently observed in prostate cancer, particularly so in mCRPC compared to other tumor types^4^. The paired deep WGS and Hi-C data are hence optimally suited for assessing the relationship between SVs and the 3D genome. As described previously, we found that SVs can be detected using Hi-C data^18,51^. Additionally, we demonstrated that interacting domains are associated with SV formation, suggesting that chromatin interactions may be a predisposing factor^52,53^, most notably between *TMPRSS2* and *ERG*, the most common recurrent gene fusion in prostate cancer. Earlier experimental work showed an androgen-induced *TMPRSS2*–*ERG* interaction and radiation-induced gene fusion in prostate cancer cell lines^54^. Hi-C studies in vitro also showed increased interactions in the region between *TMPRSS2* and *ERG* in both *TMPRSS2*–*ERG* fusion-positive and fusion-negative prostate cancer cell lines compared to benign prostate^55^, which were also observed in our mCRPC results. Interestingly, the interactions were more pronounced in the *TMPRSS2*–*ERG* fusion-positive mCRPC samples, all but one of which had WGS consistent with a monoallelic deletion. The Hi-C contact frequency in the deleted region in samples with sufficient tumor purity must therefore come from the non-*TMPRSS2*–*ERG* fusion allele^4^. This increased interaction may be a contributing factor to the development of *TMPRSS2*–*ERG* fusions. The chromatin interaction between two loci is probably an important factor in the initial formation of SVs as the ligation of two distant DNA ends after a double-strand break requires physical adjacency^52,53^. However, for an SV to grow in frequency beyond just a single cell and become detectable, it must also convey a selective advantage. Therefore, chromatin interaction is probably necessary for SV formation but not sufficient for SV clonal expansion. Thus, while the inclination toward SVs in interacting regions globally is detectable, the effect size is still modest.

Interestingly, we identified a sizeable subset (approximately one-third) of tumors with depleted *AR* locus contact across the rest of chromosome X associated with *AR* amplification, higher gene expression, ecDNA and poor response to ASI therapies. The presence of ecDNA at the *AR* locus is consistent with the large degree of *AR* amplification in mCRPC. In addition, the extent of depleted contact frequently also includes the upstream enhancer, consistent with other oncogenes^56^. Often, there was also evidence for concurrent noncircularized variants such as BFBs. BFBs and ecDNA have been reported to be present simultaneously^57^; BFBs can even result from ecDNA reincorporating into the genome^58,59^. There were a few samples where ecDNA was not identified in the WGS; however, the Hi-C patterns were suggestive of *AR* ecDNA, suggesting that Hi-C might be able to provide orthogonal information from WGS for ecDNA detection.

In addition, we observed remarkable differences in global TAD architecture between samples, distinguishing two subgroups of patients with broad versus superimposed narrow TAD patterns. Prostate cancer cell lines have been shown to have smaller TADs than benign prostate cell lines, indicating that a shrinking TAD structure may be a marker of oncogenesis^60^. We leveraged our integrated sequencing to identify that TAD-based tumor subtypes were independent of a range of biological and technical factors and were associated with orthogonal and concordant genomic, transcriptomic, epigenomic and clinical changes. The increase in local interactions coupled with the observed increase in HMRs and 5hmC are consistent with the TPM differences. Furthermore, the narrow subtype was transcriptionally enriched for expression of *MYC* targets and trended toward higher CNs of *MYC*. *MYC* is an oncogene and transcription factor known to drive proliferation and cell cycle progression in cancer^61^ through global changes in transcription factor dynamics^62^. Clinically, the narrow TAD subgroup demonstrated worse OS, which is consistent with a more proliferative and transcriptionally active state. There is evidence of *MYC*’s involvement in 3D chromatin remodeling^63^. Conversely, CTCF depletion can also affect *MYC* via loss of enhancer–promoter looping^64^.

The challenges of assembling such a large multiomic dataset include obtaining sufficient tissue, especially from small metastatic biopsy specimens, as well as the cost of deep sequencing and computing time. However, the many findings described in this study emphasize the power of combining multiple genome-wide approaches such as WGS, WGBS, RNA-seq, 5hmC-seq and Hi-C on clinical tumor samples. In addition to the insights generated in this study, we believe this dataset will be a valuable resource to the cancer and 3D genomics field.

## Methods

### Clinical cohort and Hi-C sample processing

Fresh-frozen image-guided metastatic biopsy samples were obtained from men with mCRPC, aged 45–90, as part of an institutional review board-approved biospecimen collection protocol of the SU2C/PCF WCDT consortium, as described previously^4–6,44,65^. Not all sequencing modalities were available for all patients primarily because of tissue quantity limitations, as these were small metastatic needle core biopsies and not large surgical samples. Published clinical outcome data were used for the survival analyses^44^. The specimens used in this study were frozen cryosections from mCRPC tissues. Upon thawing, these tissues were processed for Hi-C sequencing as described previously, step by step^18^. Briefly, tissue samples were thawed, fixed with formaldehyde, quenched with glycine, washed with PBS and lysed with Low-C lysis buffer supplemented with protease inhibitor with intermittent mixing. Samples were then subject to permeabilization with SDS. SDS was quenched with Triton X-100. Samples were digested with MboI and filled in with deoxyribonucleotide triphosphate and DNA Polymerase I Klenow Fragment. Samples then underwent proximity ligation and proteinase K digestion, and were de-crosslinked by overnight incubation with NaCl. Next, DNA was extracted using phenol-chloroform, precipitated with sodium acetate and ethanol, washed with ethanol and dissolved in elution buffer (QIAGEN). Next, DNA was fragmented via sonication; biotinylated fragments were pulled down using Dynabeads MyOne Streptavidin C1 beads. Finally, sequencing library preparation of the Hi-C samples was done using the SMARTer ThruPLEX DNA-seq kit (Takara Bio), amplified using PCR, followed by a double-size selection for 300–700 bp fragments using AMPure XP beads. Libraries were sent for BioAnalyzer analysis before sequencing. Libraries were then sequenced on a NovaSeq 6000 system. Benign and localized prostate cancer sample Hi-C were obtained from EGAS00001005014 (ref. ^18^).

### Hi-C data processing

Paired-end raw reads of the Hi-C libraries were processed using the HiC-Pro^66^ (v.3.0.0) Singularity container provided by HiC-Pro (https://github.com/nservant/HiC-Pro). Briefly, sequencing reads were first independently aligned to the reference human genome (hg38) using the Bowtie 2 (ref. ^67^) end-to-end algorithm and ‘-very-sensitive’ option. To rescue the unmapped chimeric fragments spanning the ligation junction, the ligation site was detected using an exact matching procedure; the 5′ fraction of the reads was aligned back to the reference genome. Unmapped reads, multimapped reads and singletons were then discarded. Each pair of aligned reads was then assigned to MboI restriction fragments. Read pairs from uncut DNA, self-circle ligation and PCR artifacts were filtered out and the valid read pairs involving two different restriction fragments were used to build the contact matrix. Valid read pairs were then binned at a 10-kb resolution. The binned contact matrix was then normalized using the iterative correction method^68^ to correct for biases such as GC content, mappability and effective fragment length in the Hi-C data.

To identify TADs, we used TopDom (v.0.0.2)^69^, which efficiently discovers boundaries between self-associating territories of chromatin. TopDom has a single parameter w, which defines the window size used to measure the degree of association between regions upstream and downstream from a bin. To optimize this parameter, we compared estimates of the Pearson’s correlation coefficient between bins defined as being within a TAD across a range of inputs and selected the w parameter that yielded the highest median correlation. We then identified A and B compartments using HiTC (v.1.34.0)^70^ with default parameters. Absent A and B calls refer to the pca.hic function returning an NA value.

On completion of iterative correction normalization and identification of the main 3D features, contact matrices were filtered to remove nonspecific ligation events, that is, low abundance pairs where no true underlying contact was present. Given that most chromosomes arrange in self-interacting territories^71^, this was defined as an order of magnitude increase relative to the average interchromosomal contact frequency (cutoff = 0.145687). The filtered matrices were then used to define contacts between gene TSS bins and enhancer bins.

### WGS data processing

Most WGS samples (*n* = 56) were previously sequenced and are available at the database of Genotypes and Phenotypes (dbGAP) (accession no. phs001648). An additional 20 samples were sequenced with WGS using the same DNA extraction and library preparation methods as for the published WGS^4^. The PURPLE tool was used to evaluate CN alterations and assess tumor purity and ploidy^72^. CN and biallelic status of the tumors were determined by incorporating tumor purity, tumor ploidy and chromosome type (autosomal or sex chromosome). Tumors were then grouped into two categories of amplified or deleted according to the following criteria. For the genes in chromosome X/Y, a tumor was marked as amplified if a minimum exonic CN was higher than tumor ploidy ×0.9. A tumor sample with deletion should have a minimum exonic CN lower than 0.75. A tumor sample with biallelic loss was required to have a maximum exonic CN lower than 0.5. For the genes in autosomal chromosomes, a tumor was marked as amplified if a minimum exonic CN was higher than its tumor ploidy ×1.95. A tumor sample with deletion should have a minimum exonic CN lower than 1.1. A tumor sample with biallelic loss was required to have a maximum exonic CN lower than 0.5. Somatic mutation analysis was performed with Strelka2 (ref. ^73^) v.2.9.10 and MuTect2 (ref. ^74^) v.3.1.0. The intersected results of both tools were chosen to improve the accuracy of the results as recommended^73^. SVs were determined using Manta^75^ v.1.6.0-3. In addition, for *TMPRSS2*–*ERG*, we also used GRIDSS v.2.12.2 and LINX v.1.17 (ref. ^76^) to confirm. Samples lacking a PASS designation were excluded from the analyses. To eliminate false positives or false negatives, the results from LINX were manually checked using IGV^77^ v.2.8.9.

For ecDNA identification, we followed the AmpliconArchitect^37^ recommendations. First, FASTQ files were realigned against the AmpliconArchitect-curated repository genome reference GRCh38 using the docker PrepareAA v.0.1203.1. Then CN calls from PURPLE (v.3.0) were smoothed using the seed_trimmer.py script from the PrepareAA tool with default parameters (--minsize 50,000 and --cngain 4.5). Then, the output BED file was fed into the amplified_intervals.py script from AmpliconArchitect (v.1.2) with optimized parameters (--gain 5--cnsize_min 100,000). Then, the AmpliconArchitect.py script was fed with the seed interval, mapped reads and parameters (--ref GRCh38--downsample -1 --extendmode EXPLORE--sensitivems False--plotstylesmall--insert_sdevs 3.0--pair_support_min 2). The classification was performed by AmpliconClassifier v.0.4.9 with the following parameters: --ref GRCh38 and --plotstyle individual--min_flow 1--min_size 5,000--decomposition_strictness 0.1. To confirm the BFB cycles, we used JaBba^78^ (v.1.1), using the Cobalt (v.1.11) ratio output, SV CN aware calls, along with purity and ploidy from PURPLE. Then, the JaBba output was imported into gGnome (v.0.1) to confirm the BFB cycle classification.

### WGBS data processing

Most WGBS samples (*n* = 55) were previously sequenced and are available at dbGAP (accession no. phs001648). An additional 14 samples were sequenced with WGBS using the same DNA extraction, bisulfite conversion and library preparation methods as for the published WGBS^5^. WGBS data were aligned to GRCh38; deduplication, then base-level methylation calling were performed using Bismark^79^ v.0.23.0 with the --pairedend and --no_overlap parameters set; otherwise, default parameters were used, as recommended by the Bismark User Guide for the library kit. HMRs and PMDs were identified using MethylSeekR^80^ v.1.22.099. Thirty percent was the threshold chosen for identifying unmethylated and low-methylated regions; otherwise, the default parameters were used. The default MethylSeekR cutoff of minimum read coverage of five or more reads was included for subsequent analysis.

### RNA-seq data processing

Most RNA-seq samples (*n* = 56) were previously sequenced and are available at dbGAP (accession no. phs001648). An additional 22 samples were sequenced with RNA-seq using the same RNA extraction and library preparation methods as for the published RNA-seq^4,5^. RNA-seq data derived from laser-captured micro-dissected samples were aligned with STAR (v.2.7)^81^. RNA abundance was calculated using the default parameters and transcripts were quantified at the gene level using GENECODE v.28, as described previously^4,5^. The expression of each gene was then converted to TPM. Mitochondrial and ribosomal RNA, and ribosomal pseudogenes, were excluded from the data.

### 5hmC-seq data processing

All 5hmC-seq samples were previously sequenced and are available at the European Genome-phenome Archive (EGA) (accession no. EGAS00001004942). Preprocessing and peak calling were performed as described previously^6^. Briefly, reads were mapped to GRCh38 and quality-filtered to remove duplicates and retain read pairs with a mapping quality greater than 30, removing orphan reads. Retained reads were used to identify 5hmc peaks in MACS2 (v.2.2.6) (p-value threshold = 0.00001) using enriched and input control samples, under the default paired-end mode, retaining only peaks in white-listed regions (chromosomes 1–22, X and Y, excluding ENCFF419RSJ^82^).

### ChIP–seq data processing

Raw ChIP–seq data for H3K27ac from primary prostate tumors and patient-derived xenografts were downloaded from the Sequence Read Archive (SRA) under accession no. SRP194063 (ref. ^10^). Reads with a base quality score greater than 30 were aligned to the human genome GRCh38/hg38 using the Burrows–Wheeler Aligner-MEM (v.0.7.17). The aligned reads were deduplicated and peaks were called using MACS2 (ref. ^83^) (v.2.2.5), with a false discovery rate lower than 0.01. Peaks falling in the genomic blacklisted regions defined by ENCODE^19^ were excluded under accession no. ENCFF356LFX. consensusSeekeR^84^ (v.1.16) was used to merge peaks in which at least two samples had one peak in the same region. As ChIP–seq was performed on different samples than our cohort, we defined a candidate enhancer in our data by an H3K27ac peak and sample-level hypomethylation^5^ present in 10% or more of our samples. ChIA–PET data were obtained from GSE54946 (ref. ^85^).

### Size-matched background for SVs

To determine the associations of SVs to Hi-C findings, we needed to generate a control size-matched background. To do this, for each sample, we took the SVs as determined by Manta. SVs were filtered to only include SVs measuring 50 Mb or less (larger SVs increased the run-time to create random SVs of a similar size) with ten or more paired-end reads and ten or more spanning reads supporting in the tumor and zero reads in the germline, respectively. We then created ten segments for each real SV, of the exact same size and type, where the start position of each was randomly identified within the genome. These random segments were not allowed to overlap gap regions (for example, centromeres, telomeres, unmapped regions).

### FISH

BAC DNA probes targeting the *AR* locus (RP11-807F19 and RP11-963N10) were labeled using a Nick Translation Kit (Abbott Molecular) with red 500 dUTP (Enzo Life Sciences), then precipitated with COT-1 DNA, salmon sperm DNA, sodium acetate and 95% ethanol. After drying, the probes were resuspended in 50% formamide hybridization buffer and combined with Abbott Molecular’s commercial probes for the X chromosome’s centromeric region. This mixture was denatured, applied to the slides with mCRPC tissue and hybridized at 37 °C in a humid chamber for 48 h. After a 2× saline-sodium citrate solution wash at 72 °C for 15–30 s, slides were counterstained with DAPI and examined for fluorescent signals using an Olympus BX61 microscope (Applied Spectral Imaging) with DAPI and fluorescein isothiocyanate filters. Images were captured with an ASI interferometer-based charge-coupled device cooled camera and analyzed using the FISHView ASI software.

### Statistics and reproducibility

Plotting and statistical tests were performed using R v.4.0.4. All statistical tests described in the article were two-sided. Box plots were generated using the R ggplot2 function (center line = median; box limits = upper and lower quartiles; whiskers = 1.5× the IQR). A two-sided Wilcoxon signed-rank test was used to assess differences between two groups. Multiple testing correction was performed using the Benjamini–Hochberg method when applicable. The box plots show the median, first and third quartiles; outliers are shown if outside 1.5× the IQR. No statistical method was used to predetermine sample size; however, our study represents the largest Hi-C study in human tumor samples, to our knowledge. No data were excluded from the analyses, the experiments were not randomized and the investigators were not blinded to allocation during the experiments and outcome assessment.

### Reporting summary

Further information on research design is available in the Nature Portfolio Reporting Summary linked to this article.

## Online content

Any methods, additional references, Nature Portfolio reporting summaries, source data, extended data, supplementary information, acknowledgements, peer review information; details of author contributions and competing interests; and statements of data and code availability are available at 10.1038/s41588-024-01826-3.

### Supplementary information

Supplementary Information Supplementary Note, Figs. 1–7 and Tables 1 and 2.

Reporting Summary

### Source data

Source Data Fig. 1 All plotted data, split into individual sheets according to the relevant components.

Source Data Fig. 2 All plotted data, split into individual sheets according to the relevant components.

Source Data Fig. 3 All plotted data, split into individual sheets according to the relevant components.

Source Data Fig. 4 All plotted data, split into individual sheets according to the relevant components.

Source Data Fig. 5 All plotted data, split into individual sheets according to the relevant components.

Source Data Fig. 6 All plotted data, split into individual sheets according to the relevant components.

## Data Availability

All mCRPC WGS, WGBS, RNA-seq and Hi-C data are deposited in the dbGAP (accession no. phs001648), EGA (accession nos. EGAS00001004942, EGAS00001006604, EGAD00001008487, EGAS00001006649 and EGAD00001009065) and Synapse (syn59759056). These Hi-C data are newly provided, whereas parts of the WGS, WGBS and RNA-seq data were previously published, with some new samples added in this study (see Supplementary Table 1 for the full details). The Hi-C matrices have been deposited in the Gene Expression Omnibus under accession no. GSE249494. Published ChIP–seq data were downloaded from the SRA under accession no. SRP194063. Blacklisted peaks defined by ENCODE are available under accession no. ENCFF356LFX. Published ChIA–PET data were obtained from GSE54946. Published benign and localized prostate cancer sample Hi-C data were obtained from EGAS00001005014. Source data are provided with this paper.

## Code availability

No custom algorithms were used, only the public data processing and visualization tools described in the Methods. Code freely available upon request.
