# Supplementary material for: Integrated analyses highlight interactions between the three-dimensional genome and DNA, RNA and epigenomic alterations in metastatic prostate cancer
Source: Nat Genet. 2024 Jul 17;56(8):1689–700. doi: 10.1038/s41588-024-01826-3 (PMC11319208; doi:10.1038/s41588-024-01826-3)
Supplement: Supplementary file 1 — Supplementary Note, Figs. 1–7 and Tables 1 and 2. [file 41588_2024_1826_MOESM1_ESM.pdf]

nature genetics

Supplementary information https://doi.org/10.1038/s41588-024-01826-3

Integrated analyses highlight interactions between the three-dimensional genome and DNA, RNA and epigenomic alterations in metastatic prostate cancer

In the format provided by the authors and unedited

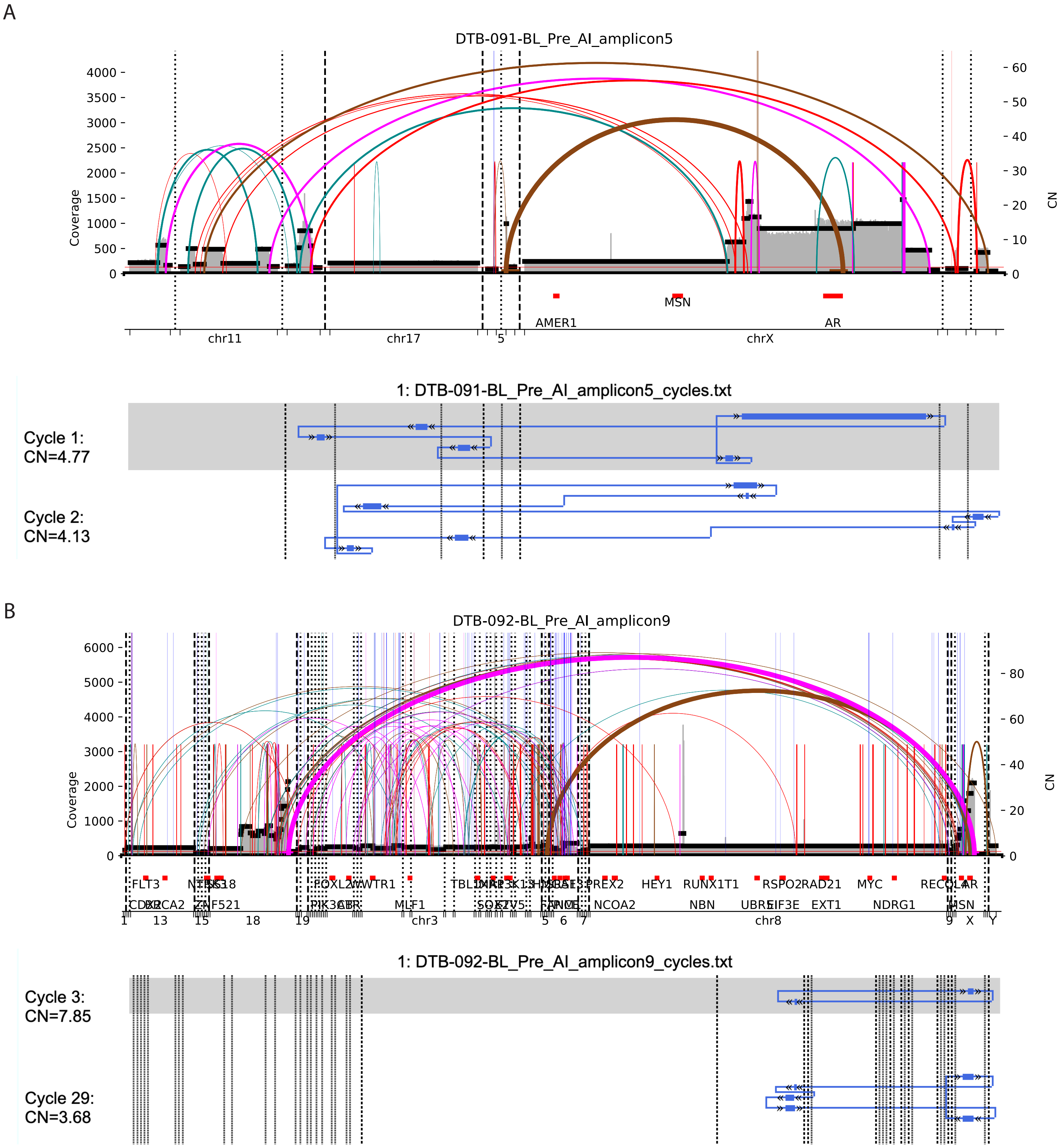

Figure S1: AmplichonArchitect results. Schematic representation of two ecDNA amplicons that co-amplified AR and segments from other chromosomes in two mCRPC tumors (**A**) DTB-091-BL and (**B**) DTB-092-BL. Both plots were generated using genomequery.ucsd.edu:8800. (**Top**) Chromosomal locations indicated and separated by vertical dashed lines. The location of genes including AR are indicated by red rectangles. Horizontal black lines indicate copy number, with scale on the right axis. Vertical grey lines indicate coverage levels, with scale on the left axis. Arcs drawn between genomic loci connect breakpoints in the amplicon, with colors linked to the orientation of the reads at the breakpoint (red: Forward/Reverse; brown: Reverse/Forward; pink: Reverse/Reverse; teal: Forward/forward). (**Bottom**) Representation of selected AmpliconArchitect cycles with overlapping segments including AR and segments in another chromosome.

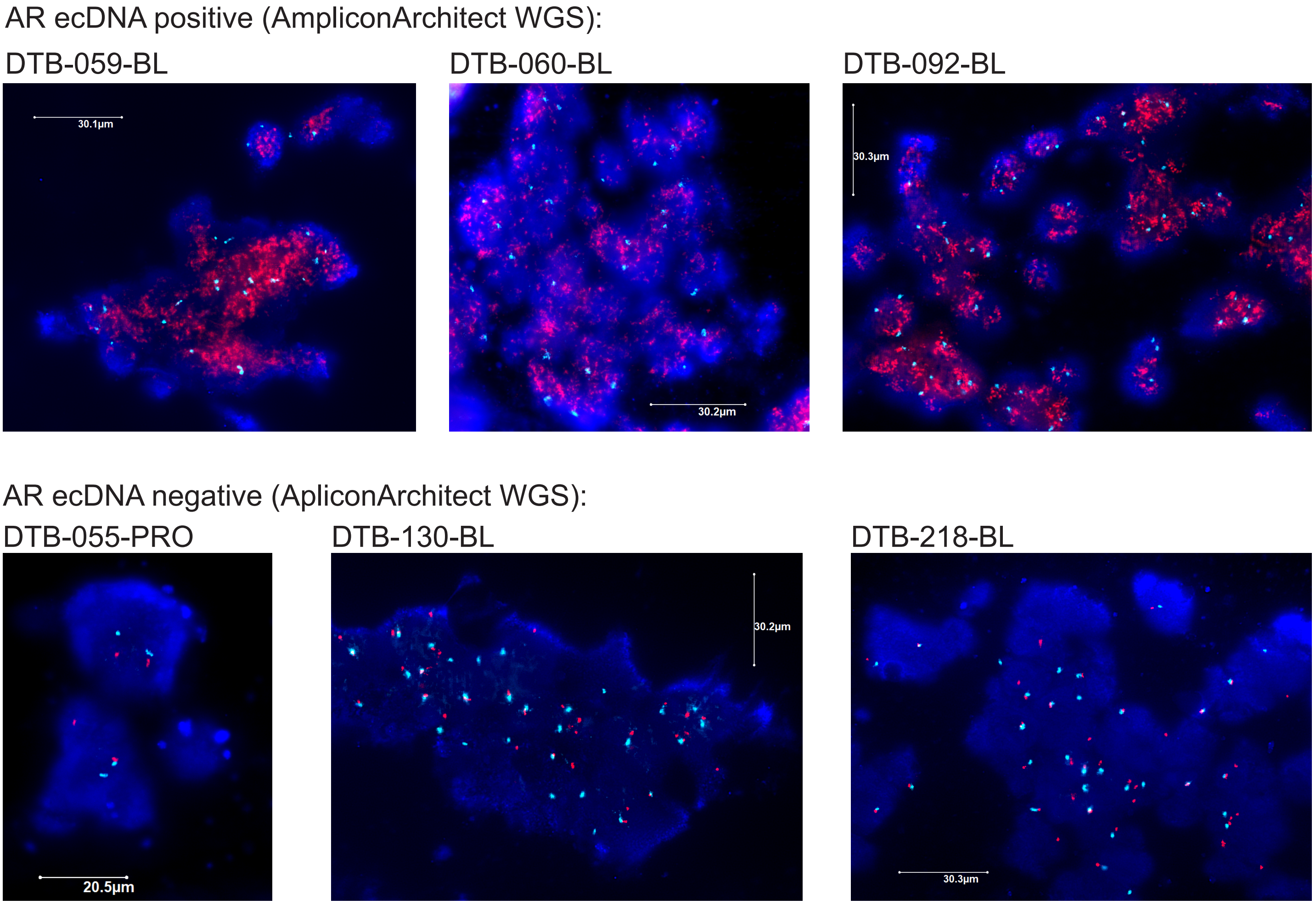

Figure S2: Fluorescence in situ hybridization (FISH) results. FISH of the mCRPC biopsy samples using fluorescence probes targeting AR (red) and chromosome X centromere (aqua), DAPI staining (blue). For ecDNA+ and ecDNA- conditions, three independent biological samples were analyzed.

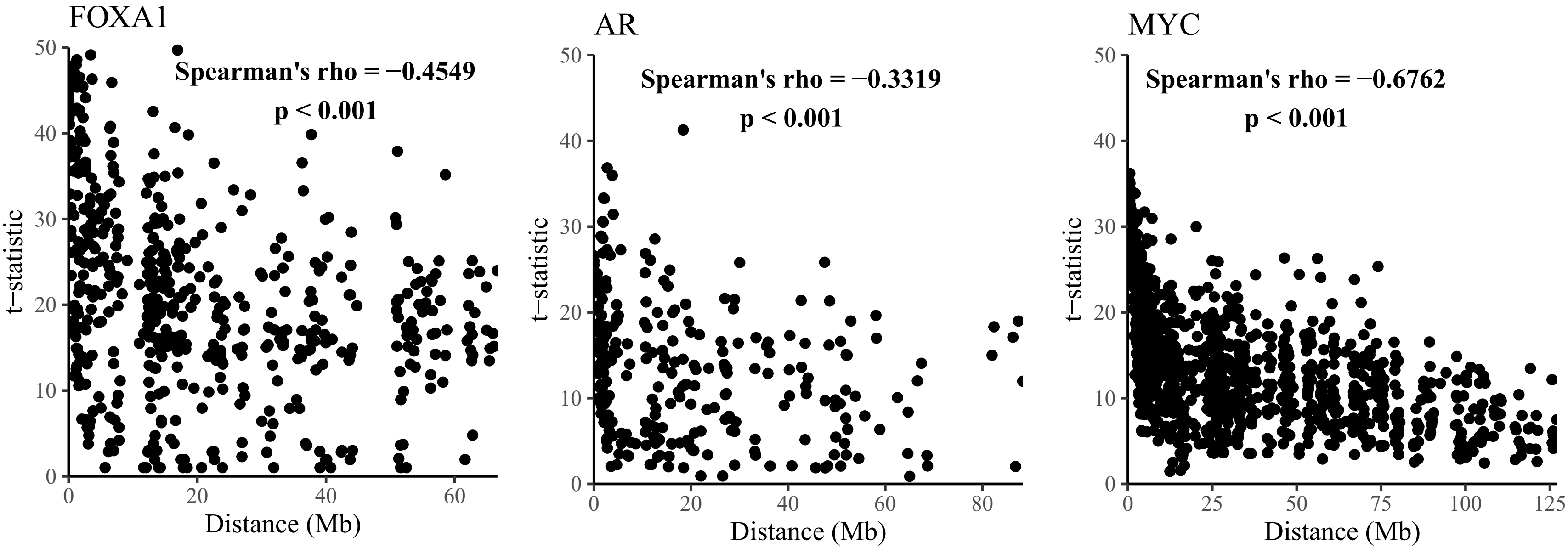

Figure S3: Gene-enhancer correlations. For all genes shown in Figure 3D-F, Spearman's correlations (two-sided) between the distance of a gene's TSS to putative enhancer contact and the t-statistic derived from the gene's TPM and methylation status at that putative enhancer. (*P* values = 5.37e-30, 2.44e-08, 1.32e-168, respectively)

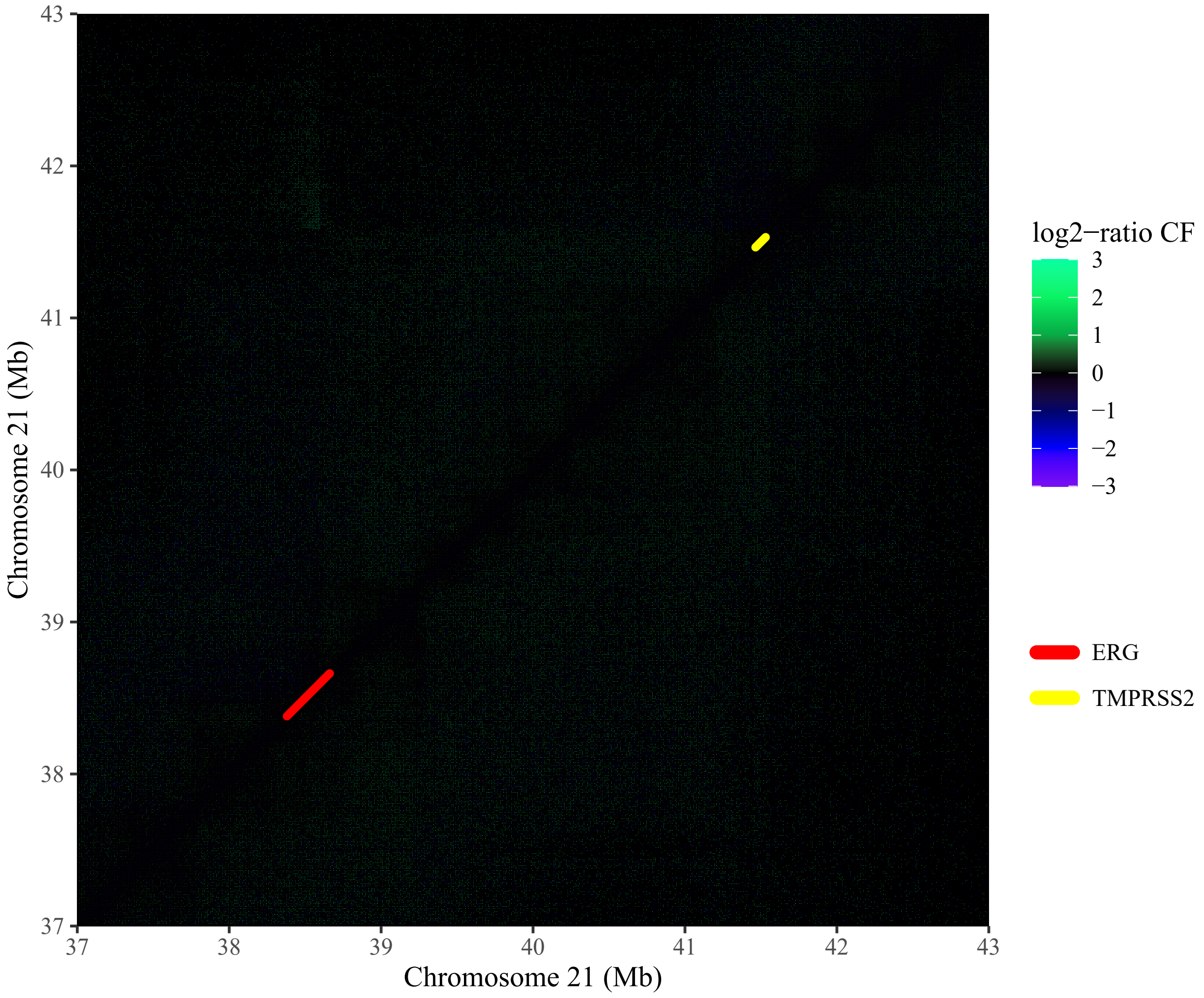

Figure S4: HiC heatmap of TMPRSS2-ERG fusion. Median centered log-ratio of Hi-C contact frequency comparing localized prostate cancer samples and benign prostate. T2E fusion-positive samples above the diagonal (canonical T2E fusion defined by WGS) and negative below. Green = higher in localized prostate cancer, blue = higher in benign prostate.

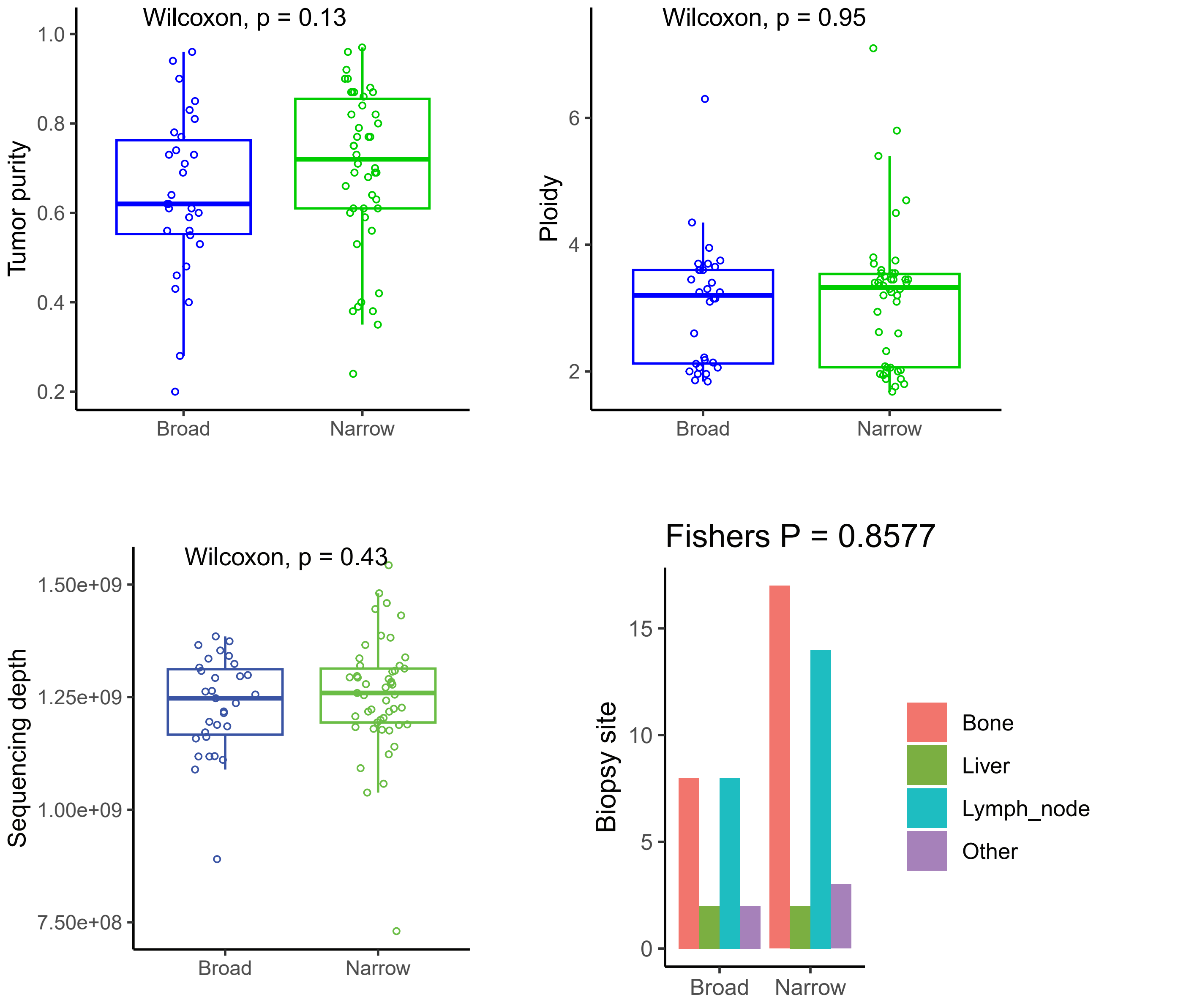

Figure S5: Comparison of subtypes across genomic and clinical features. Boxplots showing distributions between broad/narrow subtypes for tumor purity (**top left**, n = 76), ploidy (**top right**, n = 76), or Hi-C sequencing depth (**bottom left**, n = 80). Center line, median; box limits, upper and lower quartiles; whiskers, 1.5× IQR. *P* values computed from two-sided Wilcoxon rank-sum tests. Barplot of sample biopsy sites, grouped by subtype (**bottom right**, n = 80). Two-sided Fisher's exact test compared the expected vs. observed frequency of biopsy sites across the subtypes.

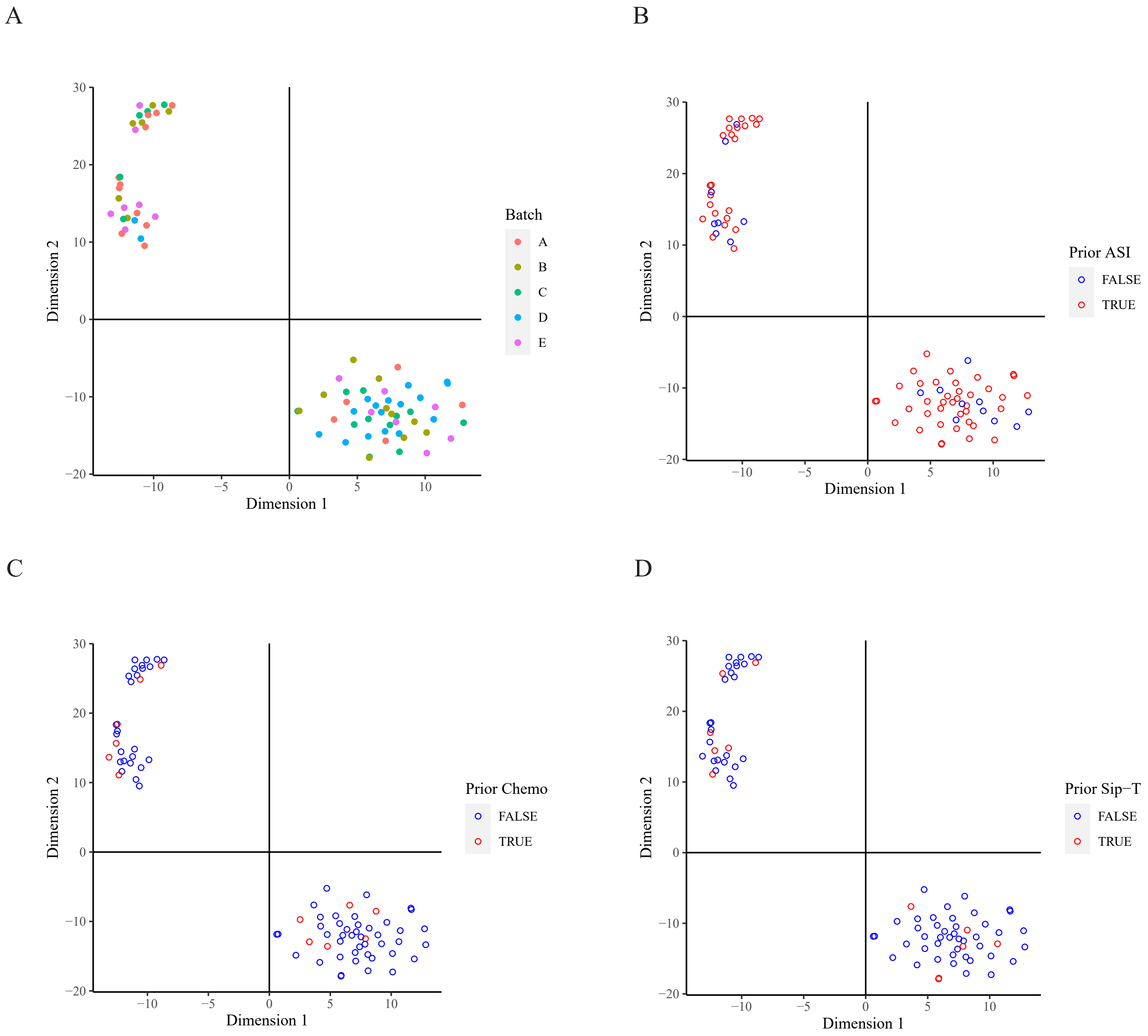

Figure S6: Subtype tSNE plots with metadata and clinical annotations. tSNE as in Figure 6A showing TAD subtypes, but colored by (A) sample processing batch, (B) ASI exposure prior to biopsy, (C) chemotherapy exposure prior to biopsy, (D) sipuleucel-T exposure prior to biopsy.

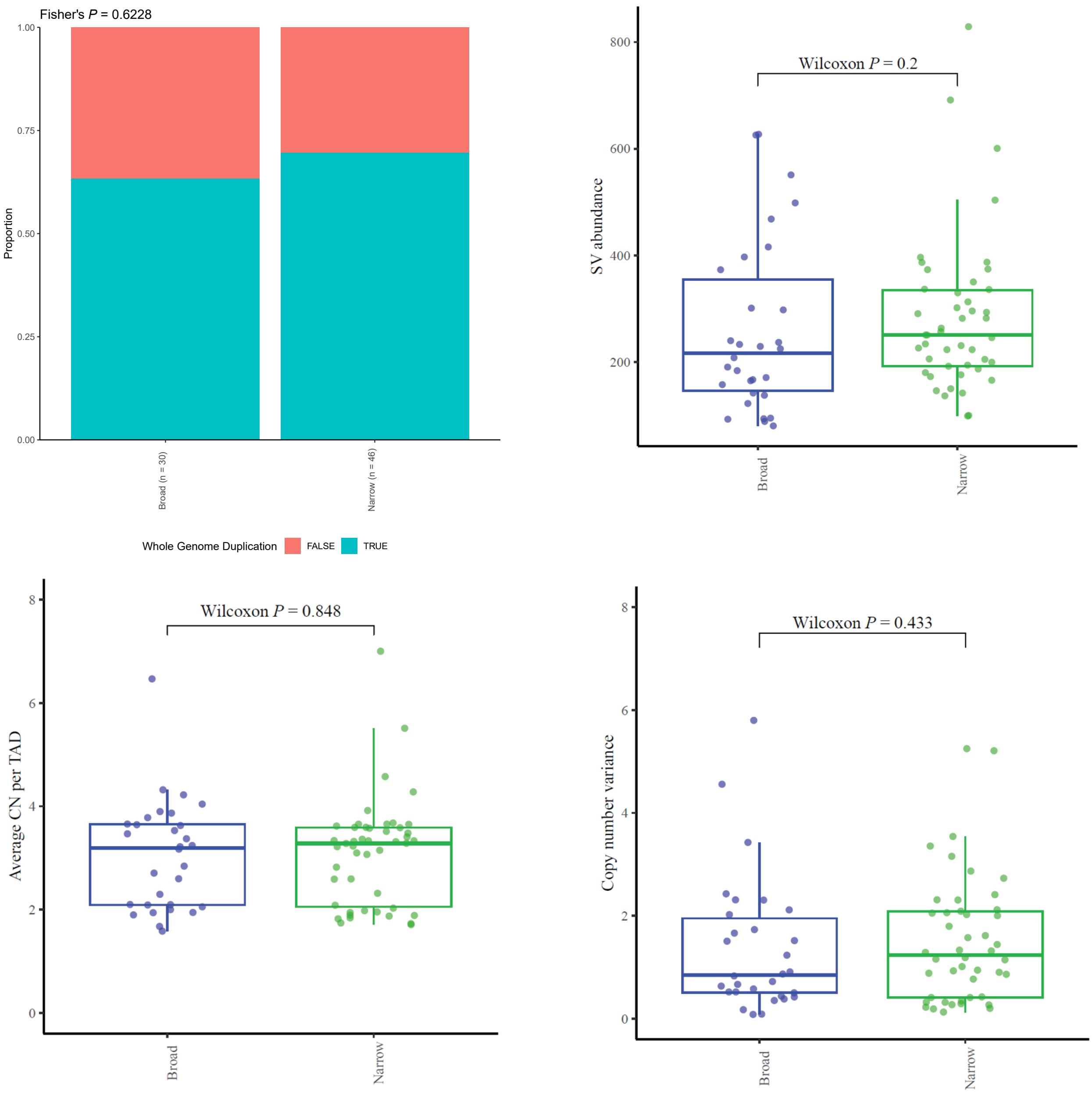

Figure S7: Subtypes compared across genomic features. Barplot of whole genome duplication, grouped by broad/narrow subtype (top left, n = 76). Two-sided Fisher's exact test compared the expected vs. observed frequency of biopsy sites across the subtypes. Boxplots showing distributions between subtypes for number of SVs (top right, n = 76), mean CN in each TAD (bottom left, n = 76), or the variance in average CN across TADs (bottom right, n = 76). Center line, median; box limits, upper and lower quartiles; whiskers, 1.5× IQR. P values computed from two-sided Wilcoxon rank-sum tests.

Table S1: Sample details, sequencing depth, and accession numbers for all samples in this study.

Sample ID	Biopsy site	HiC reads	HiC accession	RNA-seq reads	RNA-seq accession	WGS depth	WGS accession	WGBS depth	WGBS accession	5hmC reads	5hmC accession
DTB-004-BL	Other	1161589599	syn59759056	68286327	EGAD00001008487						
DTB-005-BL	Bone	1254220392	EGAS00001006604	230183968	EGAD00001008487	136X	phs001648	48X	phs001648	26106952	EGAS00001004942
DTB-008-BL	Bone	1263846858	EGAS00001006604	111176267	EGAD00001008487	125X	phs001648	46X	phs001648	23591751	EGAS00001004942
DTB-009-BL	Bone	1171782512	EGAS00001006604	112290815	EGAD00001008487	134X	phs001648	54X	phs001648		
DTB-011-BL	Lymph_node	1185173938	EGAS00001006604	93905459	EGAD00001008487	126X	phs001648	43X	phs001648	20784280	EGAS00001004942
DTB-019-BL	Other	1224164844	EGAS00001006604	121936718	EGAD00001008487	85X	EGAS00001006649				
DTB-019-PRO	Bone	1242419648	EGAS00001006604	132279841	EGAD00001008487	124X	phs001648	44X	phs001648	25772782	EGAS00001004942
DTB-021-BL	Bone	1374064803	EGAS00001006604	107173363	EGAD00001008487	138X	phs001648	46X	phs001648	22065865	EGAS00001004942
DTB-022-BL	Bone	1313444706	EGAS00001006604	76350776	EGAD00001008487	132X	phs001648	44X	phs001648	23676414	EGAS00001004942
DTB-030-BL	Other	1384891183	EGAS00001006604	62173423	EGAD00001008487	136X	EGAS00001006649	39X	EGAS00001006649		
DTB-032-BL	Other	1315565908	EGAS00001006604	44917529	EGAD00001008487	117X	EGAS00001006649	42X	EGAS00001006649		
DTB-034-BL	Lymph_node	1292653702	EGAS00001006604	83234983	EGAD00001008487	140X	phs001648	48X	phs001648	26860722	EGAS00001004942
DTB-035-BL	Lymph_node	1218123808	EGAS00001006604	111070275	EGAD00001008487	135X	phs001648	51X	phs001648		
DTB-036-BL	Lymph_node	1207432397	EGAS00001006604	130075670	EGAD00001008487	126X	phs001648	59X	phs001648	28358682	EGAS00001004942
DTB-037-BL	Bone	1298912590	EGAS00001006604	256623927	EGAD00001008487	126X	phs001648	48X	phs001648	26135635	EGAS00001004942
DTB-040-BL	Liver	1118899172	EGAS00001006604	134834009	EGAD00001008487	128X	phs001648	48X	phs001648	21982171	EGAS00001004942
DTB-044-BL	Other	1195247278	EGAS00001006604	136533376	EGAD00001008487	72X	EGAS00001006649				
DTB-053-BL	Bone	1296744660	EGAS00001006604	124616547	EGAD00001008487	139X	phs001648	49X	phs001648	32364595	EGAS00001004942
DTB-055-PRO	Other	1386570144	EGAS00001006604	333275209	EGAD00001009065	130X	phs001648			29097427	EGAS00001004942
DTB-059-BL	Liver	1365834284	EGAS00001006604	98127249	EGAD00001008487	137X	phs001648	45X	phs001648	26936242	EGAS00001004942
DTB-060-BL	Other	1188505079	EGAS00001006604	168723460	EGAD00001008487	134X	phs001648	44X	phs001648	21173345	EGAS00001004942
DTB-061-BL	Liver	1123007215	EGAS00001006604	131856383	EGAD00001008487	120X	phs001648	47X	phs001648	21867807	EGAS00001004942
DTB-063-BL	Lymph_node	1247606854	EGAS00001006604	128197928	EGAD00001008487	129X	phs001648	46X	phs001648	30102349	EGAS00001004942
DTB-064-BL	Bone	1335439331	EGAS00001006604	67083043	EGAD00001008487	140X	phs001648	49X	phs001648	24554273	EGAS00001004942
DTB-069-BL	Lymph_node	1188249812	EGAS00001006604	99037874	EGAD00001008487	126X	phs001648	47X	phs001648	23452229	EGAS00001004942
DTB-071-BL	Lymph_node	1445532468	EGAS00001006604	166396510	EGAD00001008487	117X	phs001648	49X	phs001648	31624418	EGAS00001004942
DTB-074-BL	Bone	1293420941	EGAS00001006604	282381894	EGAD00001008487	114X	phs001648	44X	phs001648	22631883	EGAS00001004942
DTB-077-PRO	Bone	1382058855	EGAS00001006604	100882762	EGAD00001008487	138X	phs001648	46X	phs001648	24287984	EGAS00001004942
DTB-080-BL	Lymph_node	1214618962	EGAS00001006604	122253765	EGAD00001008487	135X	phs001648	45X	phs001648	37912180	EGAS00001004942
DTB-080-PRO	Other	1255597587	EGAS00001006604	92799155	EGAD00001008487	109X	EGAS00001006649				
DTB-083-BL	Lymph_node	1262364838	EGAS00001006604	242900622	EGAD00001008487	134X	phs001648	49X	phs001648	22736013	EGAS00001004942
DTB-085-BL	Lymph_node	1308257115	EGAS00001006604	128488683	EGAD00001008487	137X	phs001648	45X	phs001648	27217593	EGAS00001004942
DTB-090-BL	Other	1542910084	EGAS00001006604	124482889	EGAD00001008487						
DTB-090-PRO	Lymph_node	1271490559	EGAS00001006604	128492912	EGAD00001008487	128X	phs001648	43X	phs001648	11031262	EGAS00001004942
DTB-091-BL	Bone	1290316691	EGAS00001006604	99479930	EGAD00001008487	129X	phs001648	36X	phs001648	24206842	EGAS00001004942
DTB-092-BL	Bone	1089238976	EGAS00001006604	91965829	EGAD00001008487	141X	phs001648	50X	phs001648	27445355	EGAS00001004942
DTB-097-BL	Other	1480590432	EGAS00001006604	104851565	EGAD00001008487						
DTB-097-PRO	Liver	890275599	EGAS00001006604	109458285	EGAD00001008487	126X	phs001648	46X	phs001648	27498522	EGAS00001004942
DTB-098-BL	Other	1341550353	EGAS00001006604	81321140	EGAD00001008487	148X	EGAS00001006649	40X	EGAS00001006649		
DTB-101-BL	Other	1259192946	EGAS00001006604	135964426	EGAD00001008487	127X	phs001648	43X	phs001648	23298572	EGAS00001004942
DTB-104-BL	Lymph_node	1226669866	EGAS00001006604	115643310	EGAD00001008487	132X	phs001648	45X	phs001648	50040631	EGAS00001004942
DTB-110-BL	Other	1308590870	EGAS00001006604					36X	EGAS00001006649		
DTB-111-PRO	Lymph_node	1338289319	EGAS00001006604	101336618	EGAD00001008487	138X	phs001648	50X	phs001648	26703805	EGAS00001004942
DTB-119-PRO	Bone	1277741132	EGAS00001006604	110077901	EGAD00001008487	127X	phs001648	48X	phs001648	30456653	EGAS00001004942
DTB-124-BL	Bone	1255801246	EGAS00001006604	89761284	EGAD00001008487	131X	phs001648	45X	phs001648	24287811	EGAS00001004942
DTB-125-BL	Other	1158135217	EGAS00001006604	129724942	EGAD00001008487	122X	EGAS00001006649	56X	EGAS00001006649		
DTB-126-BL	Bone	1336158083	EGAS00001006604	233754457	EGAD00001008487	125X	phs001648	47X	phs001648	20673342	EGAS00001004942
DTB-127-BL	Other	1319756562	EGAS00001006604	96526063	EGAD00001008487	115X	EGAS00001006649				
DTB-127-PRO	Lymph_node	1193774032	EGAS00001006604	107126713	EGAD00001008487	129X	phs001648	48X	phs001648	24106766	EGAS00001004942
DTB-128-BL	Bone	1217960820	EGAS00001006604	74664009	EGAD00001008487	133X	phs001648	41X	phs001648	20318651	EGAS00001004942
DTB-129-BL	Lymph_node	1057650631	EGAS00001006604	67636731	EGAD00001008487	142X	phs001648	42X	phs001648	21423182	EGAS00001004942
DTB-130-BL	Other	1458771518	EGAS00001006604	136167279	EGAD00001008487	125X	EGAS00001006649	40X	EGAS00001006649		
DTB-135-BL	Other	1365647395	EGAS00001006604	106868461	EGAD00001008487	140X	EGAS00001006649				
DTB-135-PRO	Lymph_node	1278847445	EGAS00001006604	492471208	EGAD00001008487	125X	phs001648	49X	phs001648		
DTB-138-BL	Other	1038087504	EGAS00001006604	69607708	EGAD00001008487	130X	phs001648	54X	phs001648	27936625	EGAS00001004942
DTB-140-BL	Bone	1319760056	EGAS00001006604	89886442	EGAD00001008487	136X	phs001648	50X	phs001648	30479266	EGAS00001004942
DTB-141-BL	Bone	1175836760	EGAS00001006604	333964983	EGAD00001008487	129X	phs001648	52X	phs001648	27452734	EGAS00001004942
DTB-143-BL	Bone	1293941544	EGAS00001006604	150742505	EGAD00001008487	132X	phs001648	46X	phs001648	26926634	EGAS00001004942
DTB-146-BL	Bone	1296403003	EGAS00001006604	153913814	EGAD00001008487	138X	phs001648	47X	phs001648	29424112	EGAS00001004942
DTB-148-BL	Other	1306571991	EGAS00001006604	111863135	EGAD00001008487	102X	EGAS00001006649	37X	EGAS00001006649		
DTB-149-BL	Bone	1177559080	EGAS00001006604	106907295	EGAD00001008487	121X	phs001648	46X	phs001648	26360893	EGAS00001004942
DTB-156-BL	Bone	1283909204	syn59759056	111051703	EGAD00001008487	131X	phs001648	48X	phs001648	27405628	EGAS00001004942
DTB-167-BL	Other	1353612243	EGAS00001006604	118614496	EGAD00001009065	114X	EGAS00001006649				
DTB-167-PRO	Bone	1431378697	EGAS00001006604	113643559	EGAD00001008487	133X	phs001648	54X	phs001648	28801821	EGAS00001004942
DTB-170-BL	Lymph_node	1281051291	EGAS00001006604	165811083	EGAD00001008487	118X	phs001648	44X	phs001648	29180379	EGAS00001004942
DTB-172-BL	Bone	1179696727	EGAS00001006604	129921993	EGAD00001008487	123X	phs001648	48X	phs001648	21740169	EGAS00001004942
DTB-173-BL	Lymph_node	1189377911	EGAS00001006604	120665565	EGAD00001008487	123X	phs001648	47X	phs001648	26617276	EGAS00001004942
DTB-176-BL	Other	1323773723	EGAS00001006612	91611115	EGAD00001008487	133X	phs001648	43X	phs001648	22144560	EGAS00001004942
DTB-176-PRO	Other	1236667191	EGAS00001006612			119X	EGAS00001006649				
DTB-183-BL	Lymph_node	1203814889	EGAS00001006612	94142571	EGAD00001008487	133X	phs001648	55X	phs001648		
DTB-206-BL	Lymph_node	1118167502	EGAS00001006612	126239013	EGAD00001008487	132X	phs001648	47X	phs001648	25413212	EGAS00001004942
DTB-218-BL	Other	1110826302	EGAS00001006612	92115798	EGAD00001008487	112X	EGAS00001006649	42X	EGAS00001006649		
DTB-222-BL	Lymph_node	730424302	EGAS00001006612	111732345	EGAD00001008487	130X	phs001648	54X	phs001648	27065344	EGAS00001004942
DTB-259-BL	Other	1217806998	EGAS00001006612	107519618	EGAD00001008487	113X	EGAS00001006649	41X	EGAS00001006649		
DTB-265-PRO	Lymph_node	1140044616	EGAS00001006612	137194296	EGAD00001008487	128X	phs001648	45X	phs001648	28295139	EGAS00001004942
PR-040-BL	Other	1199467302	EGAS00001006612	118037937	EGAD00001008487	101X	EGAS00001006649	44X	EGAS00001006649		
PR-056-BL	Other	1092270503	EGAS00001006612	87341149	EGAD00001008487	109X	EGAS00001006649	38X	EGAS00001006649		
PR-081-BL	Other	1222641361	EGAS00001006612	118494946	EGAD00001008487	112X	EGAS00001006649	39X	EGAS00001006649		
PR-095-BL	Other	1183530451	EGAS00001006612	153779516	EGAD00001008487	105X	EGAS00001006649	40X	EGAS00001006649		
PR-120-BL	Other	1118282130	EGAS00001006612	115700390	EGAD00001008487	104X	EGAS00001006649	45X	EGAS00001006649		

Table S2: Amplicon architect results for samples with focal amplifications at the AR locus that display detectable, complex, sub-structure. Reported for each sample is the maximum copy number of genomic segments that define the focal amplification, the proportion of segments with BFB-like characteristics, and the proportion of the genomic segments defined as: not amplified and/or invalid, non-cyclical, or cyclical in nature.

			Amplicon structure (%)		
**Sample name**	**CN**	**BFB proportion**	**No amp/Invalid**	**Non-cyclical**	**Cyclical**
DTB-060-BL	150.72	0.00	0.00	0.00	1.00
DTB-090-PRO	74.99	0.00	0.00	0.00	1.00
DTB-059-BL	72.27	0.00	0.00	0.00	1.00
DTB-069-BL	52.24	0.83	0.00	0.14	0.86
DTB-176-BL	29.56	0.00	0.00	0.00	1.00
DTB-092-BL	27.26	0.05	0.23	0.58	0.18
DTB-176-PRO	26.27	0.00	0.00	0.04	0.96
DTB-071-BL	22.47	0.58	0.08	0.04	0.88
DTB-167-PRO	19.42	0.51	0.07	0.12	0.81
DTB-135-BL	16.14	0.87	0.22	0.00	0.78
DTB-101-BL	16.31	0.76	0.28	0.00	0.72
DTB-091-BL	15.49	0.13	0.45	0.12	0.44
DTB-156-BL	13.16	0.00	0.08	0.13	0.79
DTB-265-PRO	12.38	0.62	0.34	0.00	0.66
DTB-128-BL	12.24	0.00	0.00	0.16	0.84
DTB-141-BL	10.85	0.17	0.40	0.15	0.44
DTB-126-BL	1.94	0.00	0.00	0.87	0.13
