# Supplementary material for: Integrated analyses highlight interactions between the three-dimensional genome and DNA, RNA and epigenomic alterations in metastatic prostate cancer
Source: Nat Genet. 2024 Jul 17;56(8):1689–700. doi: 10.1038/s41588-024-01826-3 (PMC11319208; doi:10.1038/s41588-024-01826-3)
Supplement: Supplementary file 2 — Reporting Summary [file 41588_2024_1826_MOESM2_ESM.pdf]

nature portfolio

Corresponding author(s): Felix Y. Feng
Mathieu Lupien
David Quigley

Last updated by author(s): 4/17/2024

Reporting Summary

Nature Portfolio wishes to improve the reproducibility of the work that we publish. This form provides structure for consistency and transparency in reporting. For further information on Nature Portfolio policies, see our Editorial Policies and the Editorial Policy Checklist.

Statistics

For all statistical analyses, confirm that the following items are present in the figure legend, table legend, main text, or Methods section.

n/a	Confirmed	
☐	☒	The exact sample size (*n*) for each experimental group/condition, given as a discrete number and unit of measurement
☐	☒	A statement on whether measurements were taken from distinct samples or whether the same sample was measured repeatedly
☐	☒	The statistical test(s) used AND whether they are one- or two-sided<br>*Only common tests should be described solely by name; describe more complex techniques in the Methods section.*
☒	☐	A description of all covariates tested
☒	☐	A description of any assumptions or corrections, such as tests of normality and adjustment for multiple comparisons
☐	☒	A full description of the statistical parameters including central tendency (e.g. means) or other basic estimates (e.g. regression coefficient) AND variation (e.g. standard deviation) or associated estimates of uncertainty (e.g. confidence intervals)
☐	☒	For null hypothesis testing, the test statistic (e.g. F, t, r) with confidence intervals, effect sizes, degrees of freedom and P value noted<br>*Give P values as exact values whenever suitable.*
☒	☐	For Bayesian analysis, information on the choice of priors and Markov chain Monte Carlo settings
☒	☐	For hierarchical and complex designs, identification of the appropriate level for tests and full reporting of outcomes
☐	☒	Estimates of effect sizes (e.g. Cohen's d, Pearson's r), indicating how they were calculated

Our web collection on statistics for biologists contains articles on many of the points above.

Software and code

Policy information about availability of computer code

Data collection

Not applicable, as no custom code or algorithms were developed to collect the data presented in the manuscript.

Data analysis

HiC-Pro (version 3.0.0)
BWA (version 0.7.17-r1198)
TopDom (version 0.0.2)
HiTC (version 1.34.0)
PURPLE (version 3.0)
Strelka2 (version 2.9.10)
MuTect2 (version 3.1.0)
Manta (version 1.6.0-3)
GRIDSS (version 2.12.2)
LI NX (version 1.17)
IGV (version 2.8.9)
PrepareAA (version 0.1203.1)
AmpliconArchitect (version 1.2)
JaBba (version 1.1)
Cobalt (version 1.11)
gGnome (version 0.1)
Bismark (version 0.23.0)
MethylSeekR (version 1.22.099)
STAR (version 2. 7)

bwa-mem (version 0.7.17)
MACS2 (version 2.2.5 and 2.2.6)
consensusSeekeR (version 1.16)

For manuscripts utilizing custom algorithms or software that are central to the research but not yet described in published literature, software must be made available to editors and reviewers. We strongly encourage code deposition in a community repository (e.g. GitHub). See the Nature Portfolio guidelines for submitting code & software for further information.

Data

Policy information about availability of data

All manuscripts must include a data availability statement. This statement should provide the following information, where applicable:

- Accession codes, unique identifiers, or web links for publicly available datasets
- A description of any restrictions on data availability
- For clinical datasets or third party data, please ensure that the statement adheres to our policy

All WGS, WGBS, RNA-seq, and Hi-C data are deposited in dbGAP (accession# phs001648) and EGA (accession# EGAS00001004942, EGAS00001006604, EGAD00001008487, EGAS00001006649, EGAD00001009065). Hi-C matrices are deposited in GEO with accession number GSE249494.

Research involving human participants, their data, or biological material

Policy information about studies with human participants or human data. See also policy information about sex, gender (identity/presentation), and sexual orientation and race, ethnicity and racism.

Reporting on sex and gender	All patients were men as we were studying prostate cancer
Reporting on race, ethnicity, or other socially relevant groupings	The vast majority of patients were caucasian with a small number of asian and african american patients.
Population characteristics	All patients were men (age 45-90) with a diagnosis of mCRPC
Recruitment	Patients were recruited by treating physicians at each contributing site in the consortium
Ethics oversight	Institutional review boards at each contributing site in the consortium

Note that full information on the approval of the study protocol must also be provided in the manuscript.

Field-specific reporting

Please select the one below that is the best fit for your research. If you are not sure, read the appropriate sections before making your selection.

☒ Life sciences ☐ Behavioural & social sciences ☐ Ecological, evolutionary & environmental sciences

For a reference copy of the document with all sections, see nature.com/documents/nr-reporting-summary-flat.pdf

Life sciences study design

All studies must disclose on these points even when the disclosure is negative.

Sample size	No sample size calculations were performed, as the Hi-C sequencing data were built upon an existing dataset of a fixed size with WGS, WGBS, and RNA-seq. This study of 80 patients with Hi-C represents the largest such study in solid malignancies to our knowledge, and thus is of a sufficient size.
Data exclusions	No data excluded
Replication	Not applicable, one sample per patient was sequenced
Randomization	Not applicable, as this was not a clinical trial
Blinding	Not applicable, as this was not a clinical trial

Reporting for specific materials, systems and methods

We require information from authors about some types of materials, experimental systems and methods used in many studies. Here, indicate whether each material, system or method listed is relevant to your study. If you are not sure if a list item applies to your research, read the appropriate section before selecting a response.

Materials & experimental systems

n/a	Involved in the study
☒	☐ Antibodies
☒	☐ Eukaryotic cell lines
☒	☐ Palaeontology and archaeology
☒	☐ Animals and other organisms
☒	☐ Clinical data
☒	☐ Dual use research of concern
☒	☐ Plants

Methods

n/a	Involved in the study
☒	☐ ChIP-seq
☒	☐ Flow cytometry
☒	☐ MRI-based neuroimaging

Plants

Seed stocks

Report on the source of all seed stocks or other plant material used. If applicable, state the seed stock centre and catalogue number. If plant specimens were collected from the field, describe the collection location, date and sampling procedures.

Novel plant genotypes

Describe the methods by which all novel plant genotypes were produced. This includes those generated by transgenic approaches, gene editing, chemical/radiation-based mutagenesis and hybridization. For transgenic lines, describe the transformation method, the number of independent lines analyzed and the generation upon which experiments were performed. For gene-edited lines, describe the editor used, the endogenous sequence targeted for editing, the targeting guide RNA sequence (if applicable) and how the editor was applied.

Authentication

Describe any authentication procedures for each seed stock used or novel genotype generated. Describe any experiments used to assess the effect of a mutation and, where applicable, how potential secondary effects (e.g. second site T-DNA insertions, mosiacism, off-target gene editing) were examined.
